# New vertical handover prediction schemes for LTE-WLAN heterogeneous networks

**DOI:** 10.1371/journal.pone.0215334

**Published:** 2019-04-17

**Authors:** Khalid M. Hosny, Marwa M. Khashaba, Walid I. Khedr, Fathy A. Amer

**Affiliations:** 1 Department of Information Technology, Faculty of Computers and Informatics, Zagazig University, Zagazig, Egypt; 2 Department of Information Technology, Faculty of Computers and Informatics, Cairo University, Giza, Egypt; Wuhan University, CHINA

## Abstract

Providing complete mobility along with minimizing the poor quality of service (QoS) is one of the highest essential challenges in mobile wireless networks. Handover prediction can overcome these challenges. In this paper, two novel prediction schemes are proposed. The first, depends on scanning the quality of all signals among mobile station and all nearby stations in the surrounding area, while the second one is based on a multi-criteria prediction decision using both the signal-to-noise ratio SNR value and station’s bandwidth. Moreover, the prediction efficiency is improved by reducing the number of redundant/ unnecessary handovers. The proposed schemes are evaluated using different scenarios with several mobile stations’ numbers, different WLAN access points, LTE-base station number & location, and random mobile station movement manner. The proposed schemes achieved a success rate of 99% with the different scenarios using LTE-WLAN architecture. The performance of the proposed prediction schemes outperformed the performance of the existing prediction schemes in terms of the accuracy percentage.

## Introduction

In the mobile environment, all users simultaneously should get full mobility while preserving the QoS where users ‘mobility significantly affects the QoS. Mobile Station (MS) must update the Base Station (BS) they are to be connected during its user movement. The execution of this process is called “handover”. MS always need to scan its surrounding users and to observe all nearby stations’ parameters such as the quality of the signal or the delay in the packet transfer to be able to execute the handover process accurately. The MS should execute the handover process to keep a continuous data transfer. Therefore, it always compares the signal parameters of the nearby serving stations with a predefined level. If the parameters of the serving station’ signal descend under these predefined values, the handover process begins to guarantee the required QoS [1].

Horizontal handover (HHO) process requires that the MS transfer its connection from the current station to another one of the same types. In this process, it requires only to re-configure the data-link layer of the Network Interface Card (NIC). On the other hand, in the vertical handover (VHO) process, the network technology might be changed by the MS. A transfer from one NIC to another and re-configuration at higher network layers are examples of these changes. In other words, the vertical handover can be defined as the ability of the MS to transfer among heterogeneous networks. Seamless handover to the best station among all existing candidates with no disruption for the ongoing data transfer, is a big challenge to achieve the user satisfaction [2].

Researchers studied handover decision strategies for a long time [1, 3]. There are several types of these strategies like conventional handover decision which normally depends on a predefined threshold of a specific parameter or values of several parameters such as power consumption, received signal strength (RSS) or the bandwidth. A vertical handover decision scheme was introduced by Savitha and Chandrasekar [4]. This scheme used a Simple Additive Weighting method (SAW) and Weighted Product Model (WPM) in selecting the best network among the existing candidate-networks to ensure unbroken connection by the MS. It uses cost, bandwidth, delay, and jitter as decision parameters.

Reducing the processing delay and introducing a trusted VHO selection from the heterogeneous wireless networks (HWNs) are the main advantages of this scheme. However, Savitha and Chandrasekar did not define a specific method to generate the weight of each individual parameter. Mahardhika et al. [5], used a number of parameters in the handover decision process where these parameters are the Received Signal Strength Indicator (RSSI), the traffic by the MS, the moving velocity and the configuration of the network. Moreover, Mahardhika and his co-authors used the network configuration as the main factor in the decision-making process.

Wang et al. presented another type of handover decision-strategy [6]. In which, the location of user movement was kept on a database that keeps-track of user movements over a long interval. This handover decision-strategy improved the MS history information which decreased the failure rate of the handover process and minimized the ping-pong handover rate. Gódor et al. [7] analyzed this handover decision-strategy and discovered that it is required a specific information about the current position of the base stations which in most cases could not be available. This is an obvious drawback.

Handover delay is one of the vital problems in wireless and mobile network communications. Remarkable attempts were done by researchers to design a suitable solution to minimize the handover delay. The redundant handover is occurred when the handover process with another station is uncompleted while the handover decision occurs. The prediction process could be helpful in solving this problem. The ping-pong impact could be defined when a handover rehashed repeatedly between two contiguous stations with a brief time-frame interim. This ping-pong impact is a kind of redundant handovers because the MS cannot exploit the association with the new station [8]. Fading impact or MS’s travel near the edges of stations are reasons of the redundant handovers. The redundant handovers cause diminishes to client’s throughput because of the administration overhead and the presented interference.

Different techniques such as Hysteresis Margin (HM) [9] and Time-To-Trigger (TTT) [10] were applied to minimize the redundant handovers number. In the HM technique, a continuous comparison between the signaling parameters of the serving station and the nearby stations, is performed where the decision and the initiation of the handover process are dependent on the results of this comparison. Once the signal strength of the predicted nearby station exceeds the signal strength of the serving station in addition to HM, the handover will be started. Other techniques such as [11, 12] were proposed to improve the operation of the handover triggering and decreasing the false handover warnings.

Predicting the next wireless network (station) with the best QoS, could be used in solving the problems of the handover delay and redundant handover. Another benefit of handover prediction is to reduce the intrusion in hard handover as well as in the situation of soft handover. When an appropriate prediction is achieved, redundant handover numbers/unnecessary handovers could be minimized. Handover prediction helps the MS in accepting the next station(s) to transfer the data connection [13–15].

Prediction schemes play an important role in mobile communications. A prediction scheme is supposed to be successful when it is able to execute a smooth handover process and keeps unbroken connectivity. Different handover decision approaches were proposed for both horizontal and vertical handover in various mobile networks. Long-Term Evolution (LTE) [16], are examples for horizontal handover while [17] are examples for vertical handover between the Worldwide Interoperability for Microwave Access (WiMAX) and LTE. The handover between two 3G, third generation of wireless mobile telecommunications technology, [18] is considered a horizontal handover while the handover between 3G and the Wireless Local Area Network (WLAN) [19] is considered an example for vertical handover.

Many handover prediction schemes were proposed. In these schemes, a balanced combination of many network’ parameters like RSS, power consumption, latency, or bit rate are used to predict the next station. The level of RSS is the most common criteria used in the prediction process due to its simplicity, easy to be measured, and its direct relationship with the QoS. The distance between the MS to its attachment point is a vital parameter in reading the RSS value. Bellavista et al. [20] proposed a scheme which uses a single threshold in prediction process. They evaluated numerous filtering methods for handover prediction by using the RSS value with Kalman filter [21], gray filter [22], particle filter [23] and Fourier filter [24]. This scheme managed the overhead and minimized the bouncing effects. Implementation of low-pass Fourier filter results in a relatively long time for mobility identification of nearby station(s) which is considered the main drawback of this scheme [25].

Becvar [26] studied the handover history in handover prediction process. In his study, Becvar tested the ratio of the predicted station against numbers of nearby stations and analyzed the impact of changing the number of stations on the success ratio. This prediction scheme is simple where any small modification of the station’ mechanism is enough for starting the prediction. The long-required time to deal with modification in the environment; unnecessary and frequent handover are the main weaknesses of this scheme. Javed et al. [27] proposed an adaptive machine learning-based boosting algorithm. This model used to examine the number of issues to predict the next handover, such as the association of the pass handoff rate (i.e. Signal Strength Variation), the size of active set (i.e. a set of stations with RSS higher than a certain threshold), and the active set update rate. This scheme works only when the information of every user is available. For example, the user’s path to the next station, his\her arrival and departure times in and out from each station in the path.

Becvar et al. [28] presented a new technique for handover prediction. This technique depends mainly on channel features such as the level of RSS for the serving and target station to improve the prediction process where this scheme required the accurate estimation of the RSS levels of all nearby stations. The handover is performed once the serving station’ RSS level becomes less than a predefined level threshold. Interruption in handovers and the signal attenuation such as shadowing and fading are the main weaknesses on this approach. Miyim et al. [29] introduced another prediction scheme which predicting the signal strength using hierarchical thresholds to choose the next target station. Significant improvement of the prediction efficiency over the other signal filtering methods is the main advantage of this prediction scheme. On the other side, this scheme couldn’t provide decent outcomes in complex environments due to ignoring the historical data.

Lin et al. [30] proposed a new vertical handover mechanism which is based on a cross-layer predictive. They used the Media Independent Handover (MIH) protocol to increase the QoS. This scheme was able to handle the connectivity issues in heterogeneous wireless networks. Reducing packet loss, optimal network selection, embedded security, reducing latency, and optimized throughput are the main advantages of this scheme. However, its drawbacks are extra signaling, context distribution, and higher resource consumption. Sadiq et al. [31] proposed a curve fitting model to correctly deduce the changing in neighboring APs‘ signal strengths. Increasing the accuracy of signal strength prediction is the main advantage of this model. Despite the fact that it improves the AP selection, it suffers from additional overhead and calculations.

Goudarzi et al. [32] proposed a new hybrid prediction scheme for predicting the next AP’ RSSI value which defined in the range of scanning area and referred as “IRBF–FFA”. This model is efficient approach where it is able to accurately predict the RSSI data. Magnano et al. [33] combined the hidden Markov models [34] and the Kalman filter model in the prediction process of this prediction handover scheme. The movement prediction and probability are used to achieve higher accuracy ratio of handover prediction.

The drawbacks of the existing handover schemes motivated the authors to propose new vertical handover prediction schemes. In this paper, the proposed two schemes offer high prediction effectiveness without demanding any signal modification or extra stresses on the MS and the network. The first proposed scheme depends on continuous reporting of the strength of the nearby network signals which scanned by the MS. In the second proposed scheme, in addition to continuous reporting of the strength of the nearby network signals, the data rate of the MS and the bandwidth available by neighboring stations, are added as effective parameters in both handover prediction and execution processes. In both proposed schemes, two heterogeneous networks are used. The Long-Term Evolution (LTE) is the base station (BS) while a wireless fidelity (Wi-Fi) which is a wireless local area network (WLAN) is the access point (AP).

The proposed prediction schemes utilized two independent thresholds. The first threshold, is defined by the signal’ strength of the present serving station while the second is obtained by the signal’ strength of candidate target station measured by the MS. Despite of the RSSI-based handover suffered from the negative effects of the Too Early, the Too Late handover and wrong selected station for handover, the proposed handover prediction scheme can overcome these problems through the proper selection of the two thresholds. These two thresholds aim at minimizing the number of redundant handovers and predict the most stable & powerful station and select it as a target station for the next handover.

The second proposed scheme utilized the signal-to-noise ratio (SNR) with the data rate of the MS, the nearby stations’ bandwidth and the level of RSSI to make the proposed scheme more practical and convenient to the environment conditions. The contributions of this paper are summarized as follows:

1. The authors proposed a new prediction handover scheme, PHO, which used the RSSI-level and the average of the two selected thresholds to perform the prediction process. The handover decision based on the level of RSSI of the target station and the Hysteresis Margin (HM).
2. The authors proposed a second PHO scheme which combines the level of RSSI, data rate needed by the MS, the bandwidth of the available target stations and SNR to predict the next station(s).
3. The proposed prediction schemes offer a list of predicted stations which increase the accuracy of the possibility of successful handover.
4. Finally, the proposed prediction schemes outperformed the existing prediction schemes using the same parameters and the environmental conditions.

The remainder of the paper is organized in five sections. A concise background of the handover types, the interconnection between LTE and WLAN, the prediction process and the relation between SNR, RSSI and data rate are presented in section 2. The detailed proposed schemes are presented in section 3. The simulation, the obtained results and the discussion are presented in section 4. A conclusion is presented in sections 5.

## Material and methods

### Handover overview

The handover process consists of five stages [35]. These are: (1) configuring the topology of the network, (2) scanning of nearby stations by MS, (3) reselecting the new cells, (4) handover decision and starting, and (5) re-connecting a new network. The initial steps (1) and (2) are executed before the process of handover. In these stages, the MS is searching through its nearby region and collecting the required data about the surrounding stations to discover the appropriate target station. The probable station is selected using the scanning process’ outcome in the structure of the cell re-selection stage.

The handover and starting decision stage will be executed once all the handover conditions are valid. At that time, synchronization with the selected station will be started by the MS. The stage of re-connecting is initiated by the MS once the synchronization is done. This stage consists of three sub-stages, the ranging stage, the re-authorization stage, and the re-registration stage. After fruitful accomplishment of every one of these three sub-stages, the MS will be able to start the handover procedure. In other words, it is able to start the data transferring using the new target station.

### Types of handover

**Fig 1:**
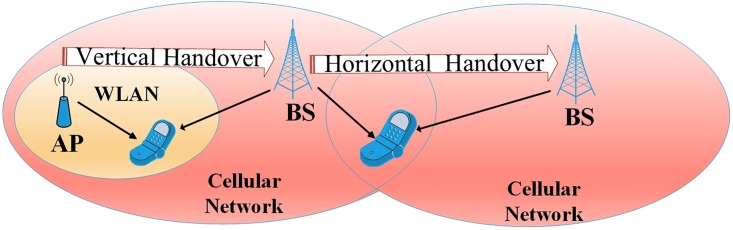
Handover types [37].

The handover process is divided into two types: The horizontal handover defined when the MS switches between stations of the same network type. The vertical handover as displayed in Fig 1 is defined when the MS switches between stations of heterogeneous networks. Smooth and effective vertical handovers is the big challenge for mobility management protocols where the existing handover schemes suffer from significant problems in keeping interconnection between the MS and the different networks.

In general, the horizontal and vertical handover could be achieved via three manners: hard, soft, and softer [36]. A hard handover is a horizontal handover with low complicated nature and simple implementation in mobile networks. Although, it causes corruption of the QoS, this handover done by cutting off the connection with the base station, before switching to another one where the MS communicates with only one station at the same times. In other words, connection with the old AP is broken before establishing the new connection.

Soft handover refers to a continuous connection between the MS and its BS until a establishing a connection with another station. In another words, the MS may be connected with two (or even more) stations at the same time. Softer handover is occurred when one BS receives 2 separated signals through multi-path propagation from different MS. These three types of handover encounter two challenges. The first one is the extra management overhead. The second one the redundant handover [38]. A successful handover prediction must overcome or minimize the negative effects of these problems.

### LTE-WLAN interworking

The mobile networks and WLANs are Internet Protocol (IP)-based systems. The architectures of these networks are not the same where the protocol stacks, the network access schemes, the mobility mechanisms, the QoS are different. Consequently, interworking between these two types of networks is not straightforward. The I-WLAN [39] is the first protocol designed to manage the interconnection of mobile networks and the WLAN networks. The I-WLAN is designed with 3^rd^ Generation Partnership Project (3GPP) mobile architecture. The architecture of I-WLAN defined the confusion among these networks in terms of data, control behaviors, access protocols, and authentication procedure. This architecture is able to handle the untrusted WLAN networks, which is a form of WLAN that is not controlled by the cellular provision supplier such as Corporate WLAN.

**Fig 2:**
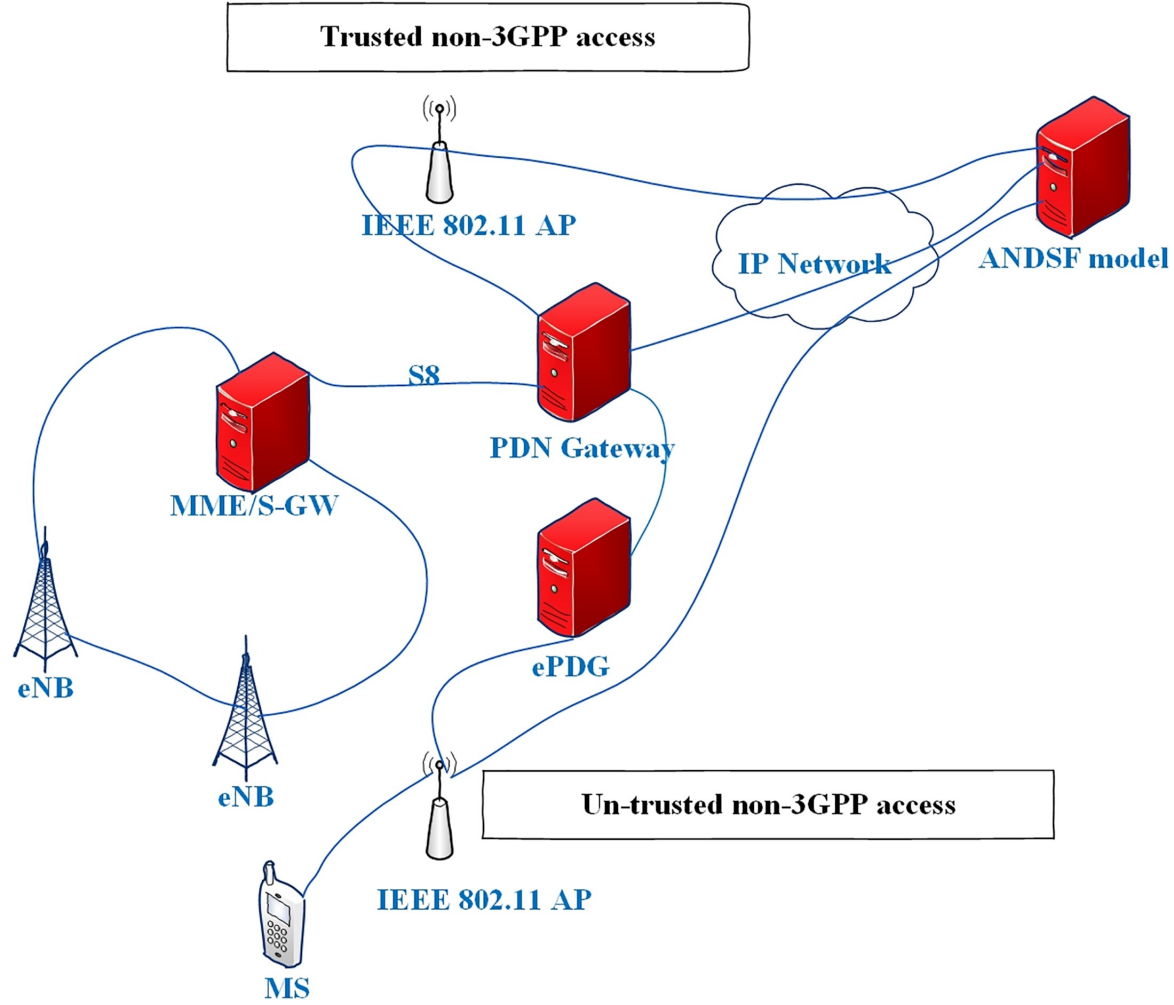
LTE-WLAN interconnection [41].

Evolved Packet Core (EPC) is presented in Release15 [40], which characterized interconnecting functionality between 3GPP and non-3GPP access systems. It became conceivable to have new choices for mobility using inter-technology handover above numerous access network systems. Moreover, a trusted WLAN network is presented in EPC, which facilitates smooth handover between 3GPP and non-3GPP networks as shown in Fig 2.

### Prediction-based handover

The target station prediction depends on few parameters such as handover history, client’s development direction, and the radio channels quality. The primary way is to store the identified data from previously completed handovers with all MSs in the network.

Serving and target stations must be updated with the essential information such as MS’ location, nearby stations and the signal strength which gathered from the MSs in the network. The secondary way to prediction the next handover is based on previous serving station. To achieve a successful prediction of the next MS’ location and movement; Three requirements should achieved [42]:

1. Accurate determination of the network domain and the present and the previous locations of the MS. Global Positioning System (GPS) is an obvious example [43].
2. Prior knowledge of the MS’ profile such as most loved spots and time plan and secured MS’ data.

The profile of the region where the prediction is performed must be identified. Appropriate geographic zones guide must be available.

Most existing movement-based prediction schemes required the RSS level, the speed of the mobile station as well as the mobile direction or a combination of both. The existing channel features-based prediction schemes use the transacted data between the MSs and the basic network. The remarkable studies [6, 20, 26] clearly show that the channel features-based prediction schemes are better than the history-based handover schemes. However, the channel features-based prediction schemes depend on the MSs ‘locations.

### The relation between SNR, RSS and data rate

The SNR could be defines as the dimensionless ratio of the signal power to the noise power in the scanning process [44]. The SNR usually used to measure the performance ratio of the signal processing structures in the presence of the Gaussian noise. In practice, a minimum value of RSSI = -65 dBm (decibel-milliwatts) which equivalent to SNR = 20 dB for a noise floor = -85 dBm. The SNR is calculated using the following form:

$$SNR=\frac{TransmitPower*ChannelGain}{NoisePower}$$ (1)

The 802.11 standard [45] used in determining the minimum values of SNR needed for the right decoding of each data rate as shown in Table 1. The bandwidth of the nearby station could be estimated using the measured values of the SNR which enable designers of the wireless networks to reduce the number of utilized units. During the last decade, the RSSI or the SNR values was used as a manufacturing standard for the wireless networks. Currently, the data rate is the alternative manufacturing standard. The conversion provides the designer with a maximum reliability and accessibility through the planning procedure.

**Table 1:** Data rates, SNR and RSS [44].

**Rate (Mb/s)**	1	2	5.5	11	6	9	12	18	24	36	48	54
**SNR (dB)**	4	6	8	10	4	5	7	9	12	16	20	21
**Signal level (dBm)**	-81	-79	-77	-75	-81	-80	-78	-76	-73	-69	-65	-64

**Fig 3:**
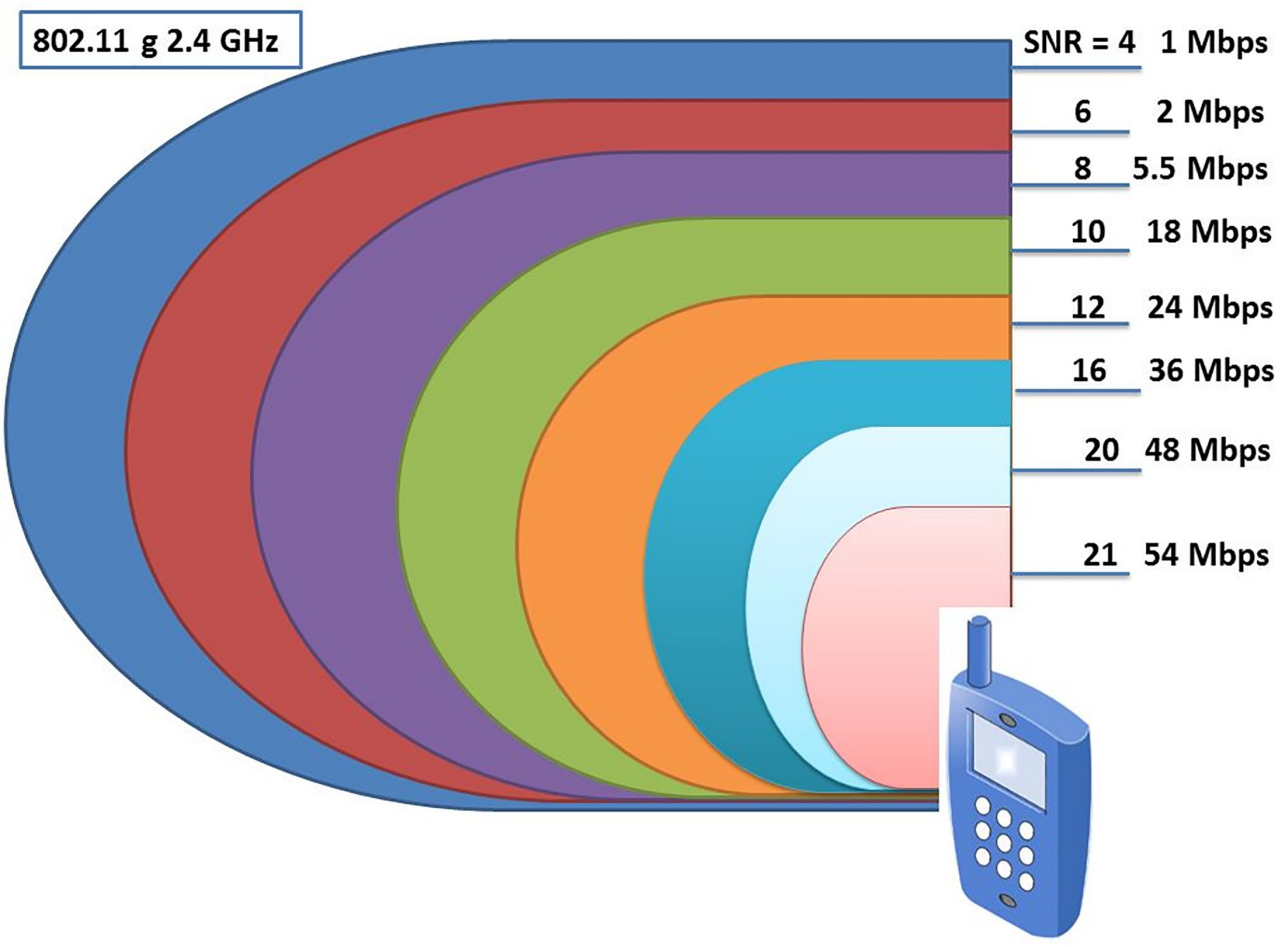
SNR and data rate for 802.11 g.

Fig 3 illustrates the equivalent relationship between the SNR and the data rate. The actual distance (radius of the circle cantered by the AP) between the AP and each MS-SNR or MS-data rate might differ with the actual effective isotropic radiated power (EIRP) of the AP. The information about the RSS level and the receivers’ noise power is required to compute the SNR value. Since the power recourses of the MS are limited, it is preferable to divide the load of measuring the RSS values between the MS and the wireless sensor network (WSN).

The bandwidth of each station could be determined using the proportional fairness scheduling technique [46]. Generally, scheduling algorithms as reported in [47] are used to reduce the negative effects of delay, packet loss rate, throughput, and delay jitter in order to improve the QoS. The scheduler needs certain information to produce correct scheduling decision like sessions’ reserved rates, link conditions and their number, and the session queues’ statutes.

## Proposed handover prediction schemes

In this section, all details of the two proposed prediction schemes are presented. The first proposed prediction scheme is called “RSSI-based handover prediction scheme”. The second prediction scheme is called “Multi-criteria handover prediction scheme”.

### RSSI-based handover prediction scheme

Recently, the RSSI is used as measure of the radio connection quality. This measure is based on the RSS, noise and the power of the AP. This indicator helps the MS to select the suitable wireless application protocols (WAPs) for offloading. The connected LTE measures the RSSI of the WLAN and compared it with the predefined threshold. The MS is connecting with the AP of the highest RSSI which must be greater than the threshold. Otherwise, the MS keeps connecting to the LTE network.

The proposed prediction scheme is based on continuous reporting of the incoming signal quality. The MS performs the scanning process using the Orthogonal Frequency Division Multiplexing (OFDM) technology which supports continuous hand-check for all available stations when the Wi-Fi is open. The energy might be wasted by the MS which having more than one station in its neighboring area. Ideal listing, collisions, frame errors, protocol overhead, overhearing, and traffic fluctuations are the main causes of this energy wasting. Therefore, higher RSSI reduced the frame errors, provide a higher performance and save energy. Tuysuz and Uçan [48] showed that, a connection of an MS to the AP with a higher RSSI results in an optimal energy saving, a complete description of scanning process for android-based mobile devices, increasing the overall throughput and providing a better service quality. Hence, the RSSI prediction-based schemes minimally affect the battery life.

The proposed prediction schemes are depending on two new thresholds. The first and the second thresholds are called (*HO*_*thrSer*_*x*,*y*_) and (*HO*_*thr Pred*_*x*,*y*_) respectively. The value of the first threshold is determined by the RSSI level of the serving station at the moment of the MS handover to the target station while the value of the second threshold is determined by the RSSI level of the predicted target station at the moment of MS handover. The values of these thresholds are not equal due to of the non-stationary signal levels. Although, the difference between the values of these two thresholds is small, these thresholds are not equivalent.

The decision of handover is normally done if the target station is able to offer better quality of connection than the current serving station. In this case, the value of the threshold, *HO*_*thr Pred*_*x*,*y*_, is considerably greater than the corresponding values of the threshold, *HO*_*thrSer*_*x*,*y*_.

If (the RSSI level of the serving station, *BS*_*x*_/*AP*_*x*_, is less than the threshold, *HO*_*thrSer*_*x*,*y*_)

&&

  (the RSSI level of the nearby station, *BS*_*y*_/*AP*_*y*_, exeeds the threshold, *HO*_*thr Pred*_*x*,*y*_,)

Then:

  MS handover from *BS*_*x*_/*AP*_*x*_ to *BS*_*y*_/*AP*_*y*_.

Where the subscripts *x* and *y* refer to the serving and target stations respectively.

To perform the prediction, we used what is called Handover zone (*HO*_*Zone*_) with both thresholds. It is the interval when a station, *BS*_*y*_/*AP*_*y*_ is marked as the candidate target station. The value of the first threshold is added to the *HO*_*Zone*_ according to Eq (2) to give the MS enough time to prepare the prediction list. The predefined *HO*_*Zone*_ is subtracted from the second threshold according to Eq (3) to select the candidate station.

$$HO\_thrSer_{x,y}+HO_{Zone}>RSSI_{BS_{x}}$$ (2)

$$HO\_thr\text{Pr}ed_{x,y}-HO_{Zone}>RSSI_{BS_{y}}$$ (3)

Where $RSSI_{BS_{x}}$ and $RSSI_{BS_{y}}$ refer to the RSSI for serving and target stations respectively.

The values of the two thresholds, *HO*_*thrSer*_*x*,*y*_ and *HO*_*thr Pred*_*x*,*y*_, are varying with each handover process between the serving and the target stations. It is better to compute the average values for both thresholds and use these averages as reference values for both thresholds. The following equations define the average thresholds.

$$avgHO\_thrSer_{x,y}=\frac{1}{HO_{BS_{x},BS_{y}}}\sum_{i=1}^{HO_{BS_{x},BS_{y}}}{RSSI}_{{BS}_{x}}$$ (4)

$$avgHO\_thrPred_{x,y}=\frac{1}{HO_{BS_{x},BS_{y}}}\sum_{i=1}^{HO_{BS_{x},BS_{y}}}{RSSI}_{{BS}_{y}}$$ (5)

**Fig 4:**
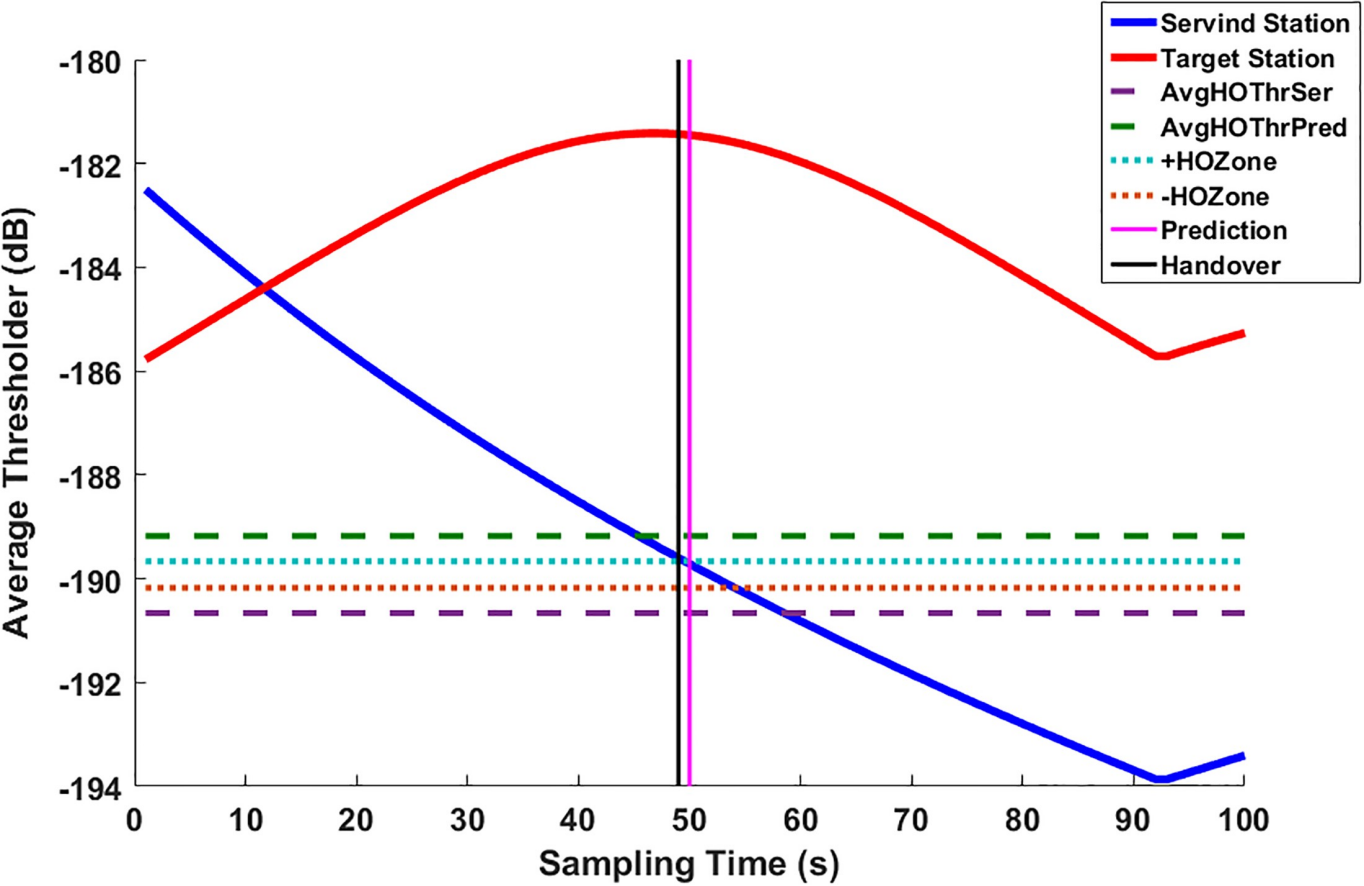
Signal RSSI quality scanned by an MS.

Where $HO_{BS_{x},By}$ is the number of handovers occurred between the current serving station, *BS*_*x*_/*AP*_*x*_ and the target station, *BS*_*y*_/*AP*_*y*_ through the observed time period. Fig 4 displayed the values of the RSSI of both serving and target stations in the scanning process.

The MS may reach to an area where more than one possible target station meets the prediction conditions. The proposed prediction scheme selects the most probable target station, *BSy*/*APy* based on the computation of the most minimal contrast between the thresholds, *HO*_*thrSer*_*x*,*y*_ & *HO*_*thr Pred*_*x*,*y*_, and the $RSSI_{BS_{x}}$ and $RSSI_{BS_{y}}$ values using the proposed Eq (6). This process is performed for all possible target stations where these stations are recorded in *ListPred*(*BS*/*AP*).

$$Diff(BS_{x},BS_{y})=|avgHO\_thrSer_{x,y}-RSSI_{BS_{x}}|+|avgHO_{thr\text{Pr}ed_{x,y}}-RSSI_{BS_{y}}|$$ (6)

The, *Diff*(*BS*_*x*_, *BS*_*y*_), for all possible target stations are sorted in one list where Eq (7) used to define the predicted station, *BS* / *AP*, as the station with the minimum difference value.

PredTar(BS/AP)=min(ListPred(BS/AP)) (7)

**Fig 5:**
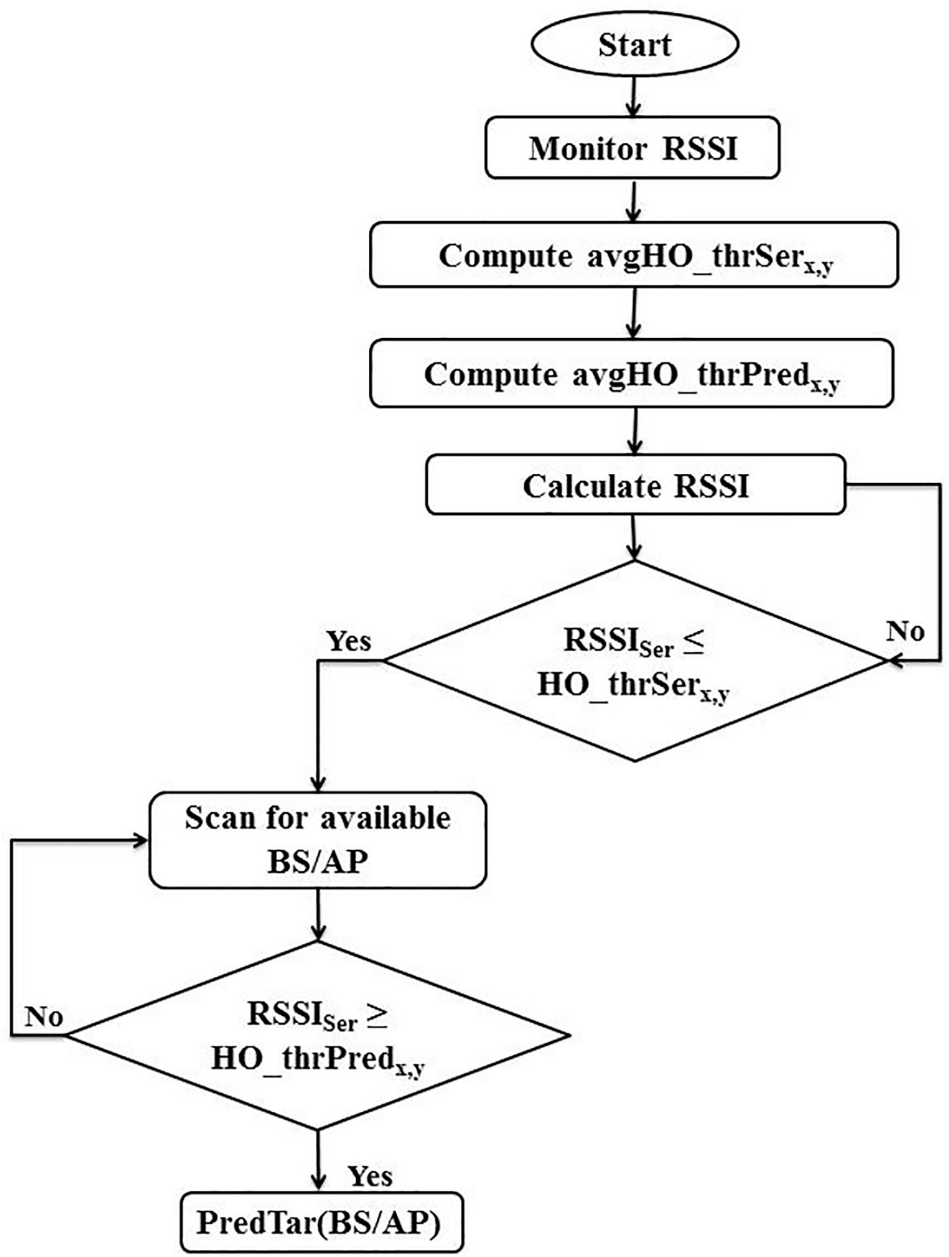
Proposed RSSI-based prediction handover scheme.

All steps of the proposed Prediction Handover scheme (PHO) are summarized and illustrated in Fig 5.

### Multi-criteria handover prediction scheme

The multi-criteria handover prediction and decision scheme are divided into two successive stages. The first stage is devoted to the handover prediction scheme while the second stage is devoted to the handover decision scheme. The handover prediction scheme is based on the determination of the SNR and the bandwidth of the nearby candidate stations. The target wireless technology-based station must have a maximum value of the SNR and a maximum bandwidth especially at the boundary of the station domain. In this scheme, the network/environment parameters are determined where continuous scanning of the RSSI level for both serving and all nearby stations is performed. The SNR values for different stations are determined. The MS used the determined SNR of each candidate nearby station to determine the data rate.

The proposed prediction scheme detects the available bandwidth of all nearby stations to decide if the station has enough bandwidth or not. Stations with enough bandwidth are suitable for possible handover while the stations with small bandwidth are marked as blocked stations where these stations are not suitable for handover. The proposed prediction scheme defined a new parameter called actual data rate (Arate). The value of this parameter is calculated the following equation:

Arate=min(SBW,MSrate) (8)

Where ***SBW*** refers to the bandwidth provided by the neighboring station(s) and ***MSrate*** is the data rate needed by the MS to establish a stable connection.

A full description of the proposed multi-criteria prediction scheme is summaries through the following steps:

1. Continuous scanning and reporting of the RSSI level for both serving and all nearby stations.
2. If the RSSI value of the serving station is less than the value of the first threshold, the prediction process will be initiated.
3. Stations with acceptable RSSI values are arranged according to the power level of each station.
4. Compute the SNR value with acceptable RSSI values.
5. Compute the MS data rate.
6. Determine the bandwidth for stations with acceptable RSSI values.
7. Compute ***Arate*** which is the main factor to decide the predicted station(s).
8. Create the prediction list according to the values of ***Arate***.

The prediction stage is completed by performing these 8 steps.

**Fig 6:**
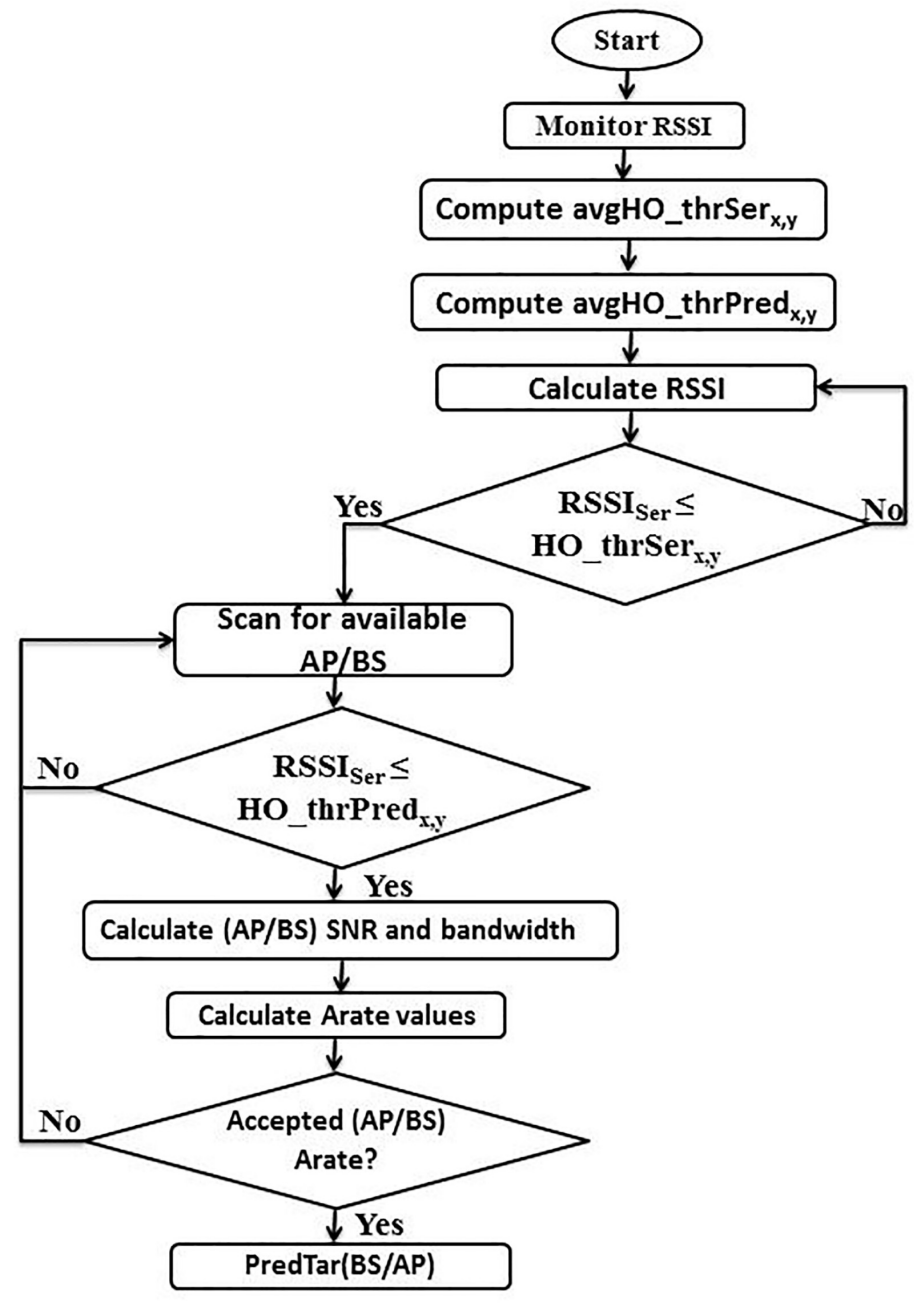
Proposed multi-criteria prediction scheme.

By completing the handover prediction process in the first stage, the handover decision in the second stage is started when the RSSI value of the target stations exceed the value of the second thresholds, ***HO*_*thr Pred***_***x*,*y***_. The decision of selecting the target station depends on the ***Arate*** value which is recalculated in the handover decision stage. Fig 6 gives an overview illustration of the proposed multi-criteria prediction and decision scheme.

The problem of losing energy and saving the MS battery life is an additional challenging problem especially with frequent MS transfer (send/receive) data. To overcome this problem, short time slots are devoted to transfer data. The proportional fair scheduling technique [46] is used to determine the proper time slots and avoid the negative effects on the QoS.

The proposed handover prediction scheme updates the prediction list to improve the success ratio (SR) percentage. The SR is defined as the ratio of the number of successful predictions to the total number of handovers. In the proposed prediction scheme, few stations with the best power level and the highest ***Arate*** values are selected in order to reduce the mobile battery usage, reducing the power needed to establish the connection to the predicted stations, and to decrease the overhead on to the LTE network.

The performance of the proposed prediction handover schemes is evaluated using the following parameters:

- The successful ration (SR),
- The time between the prediction and the handover (PHO-Time),
- The effect of MS’ speed along with the prediction accuracy,
- The number of handovers with and without the proposed prediction schemes.

The number of successfully predicted handovers (***SP***) is used to compute the ratio of the prediction success using the following formula:

$$SR=\frac{SP}{N_{HO}}\;0\leq SR\leq 1$$ (9)

Where *N*_*HO*_ refers the total numbers of the handovers in the predefined time period. The *N*_*HO*_ could be computed using the following equation:

$$N_{HO}=\sum_{BS_{x}=1}^{N_{BS}}\sum_{BS_{y}=1}^{N_{BS}}HO_{{BS}_{x},BS_{y}}\;BS_{x}\neq BS_{y}$$ (10)

Where *N*_*BS*_ is an overall stations number in the network. Prediction error (wrong prediction) occurs if the MS handover to a station which is not in the prediction list.

## Results and discussion

**Fig 7:**
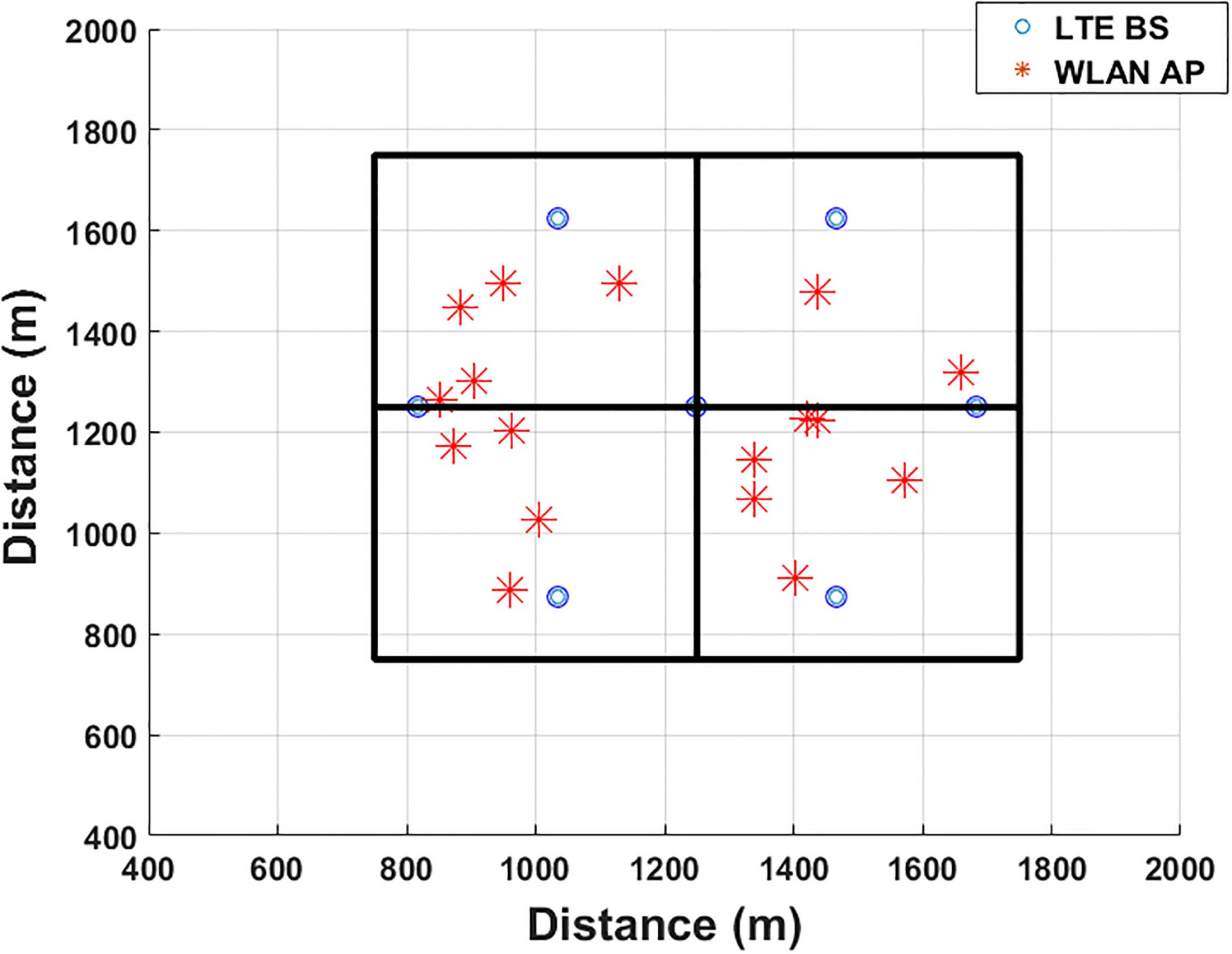
Simulated environment.

The area for simulation is divided into four sub-areas as displayed in Fig 7. In each sub-area, different APs and different MSs with various speeds and locations are used in our simulation. We utilized the random waypoint mobility model (RWPMM) [49] as a movement manner of all MS. By comparing the PRWMM with other mobility models, it is observed that the PRWMM provide the highest level of movement’ randomness which make the simulation very realistic. The average speed of a normal human is 5 km/hour [50] which equivalent to 1.38 m/s. In the simulation we used a MS speed in a random interval from 0.5 m/s to 3 m/s. The suggested lower limit, 0.5 m/s, is suitable for elderly people while the suggested upper limit is suitable for people with swift movements. The proposed handover prediction schemes are evaluated by using the MATLAB16 within different areas. The positions of all MSs and *APs* are produced in a random manner.

### Simulation scenario

The LTE Base stations are organized in a symmetrical way using the same height and their transmitting power are at the same level. The WLAN access points are organized in a random manner at the same height and the same transmitting power. There are a number of parameters the affect in the process of network selection during vertical handover such as MS’ speed, the RSSI level of the existing/target stations, the energy required for scanning various networks through different interfaces, the required time for the network connection.

Various values of simulation parameters and their ranges are shown in Table 2. The RSSI level for different MS and the nearby stations, *BS*/*AP*, are calculated using the urban macrocell path loss model [51]. The average values of the thresholds, *avgHO*_*thr ser*_*x*,*y*_ and *avgHO*_*thr Pred*_*x*,*y*_, are calculated from monitoring the first 200 sample in each area to be able to generate handover map for all station (*BS* /*AP*) in the simulated areas. Initially, we run the simulation without any predicted condition, then the handover to the station with the largest RSSI level is started.

**Table 2:** Simulation parameters.

Parameter	Value
Number of *BS*_*s*_	19
Number of *AP*_*s*_	9, 12, 15, 17
Number of *MS*_*s*_	45, 60, 70, 90
*BS* transmitting power [dBm]	46
*AP* transmitting power [dBm]	46
*MS* speed [m/s]	0.5–3
*MS* gain [dBm]	10
*BS* Frequency band [GHz]	2.5
*AP* Frequency band [GHz]	2.4
Simulation duration [s]	10800
Scanning reporting period [s]	1
path loss model	Urban Macrocell [51]
mobility model	PRWMM
Hysteresis margin HM [dB]	1–5

**Fig 8:**
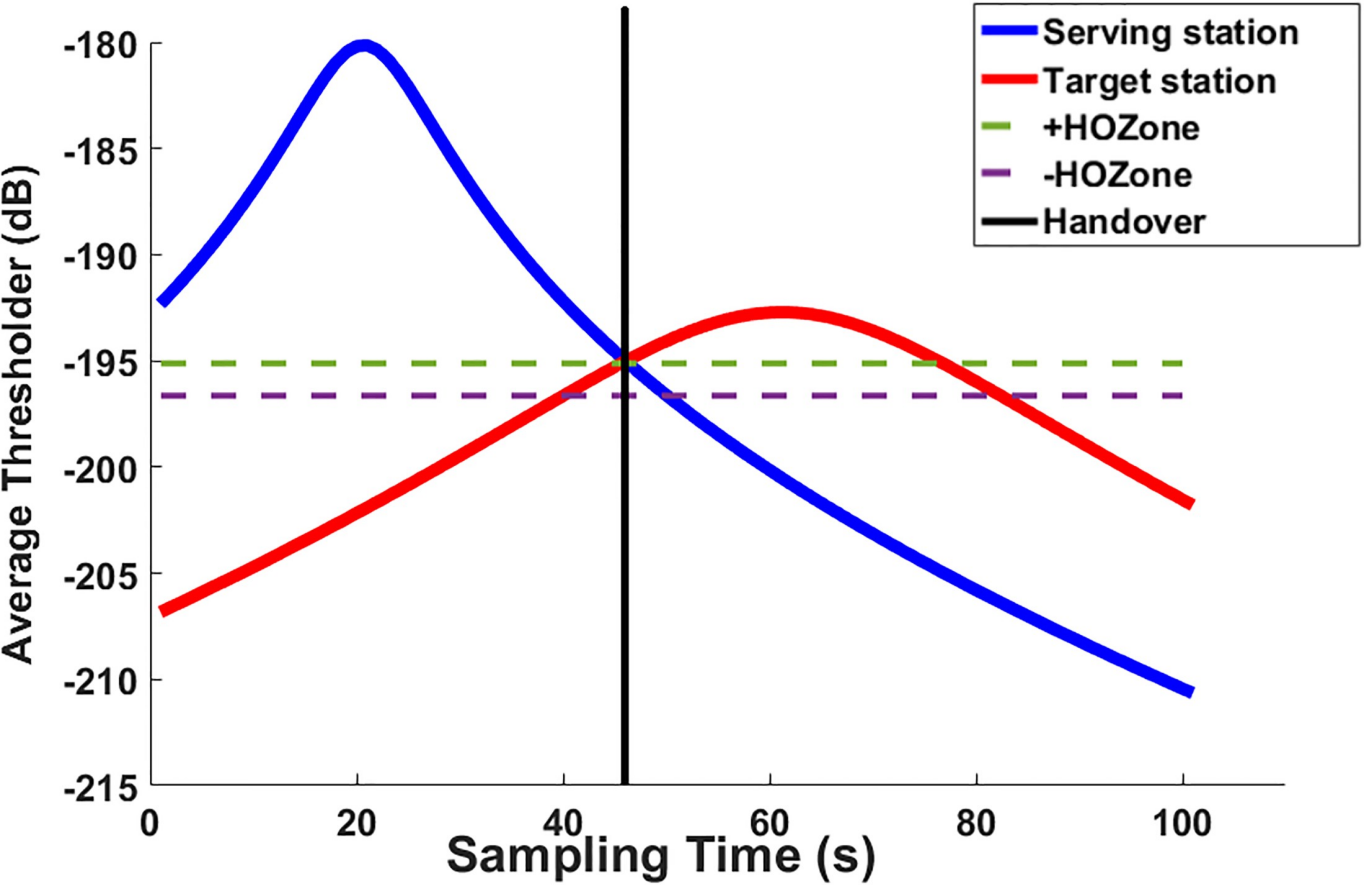
Normal handover.

This process is illustrated Fig 8. In this figure, two curves of the RSSI for serving station (blue color) and the predicted station (red color) are displayed. The handover occurred once the RSSI level of the predicted station is greater than the RSSI level of the serving one.

Although initiating the prediction process in both schemes are similar, there are few differences in performing the selection of the predicted station(s) and to which station the handover process will be occurred. These differences are summarized in the following subsections.

#### RSSI-based prediction

**Fig 9:**
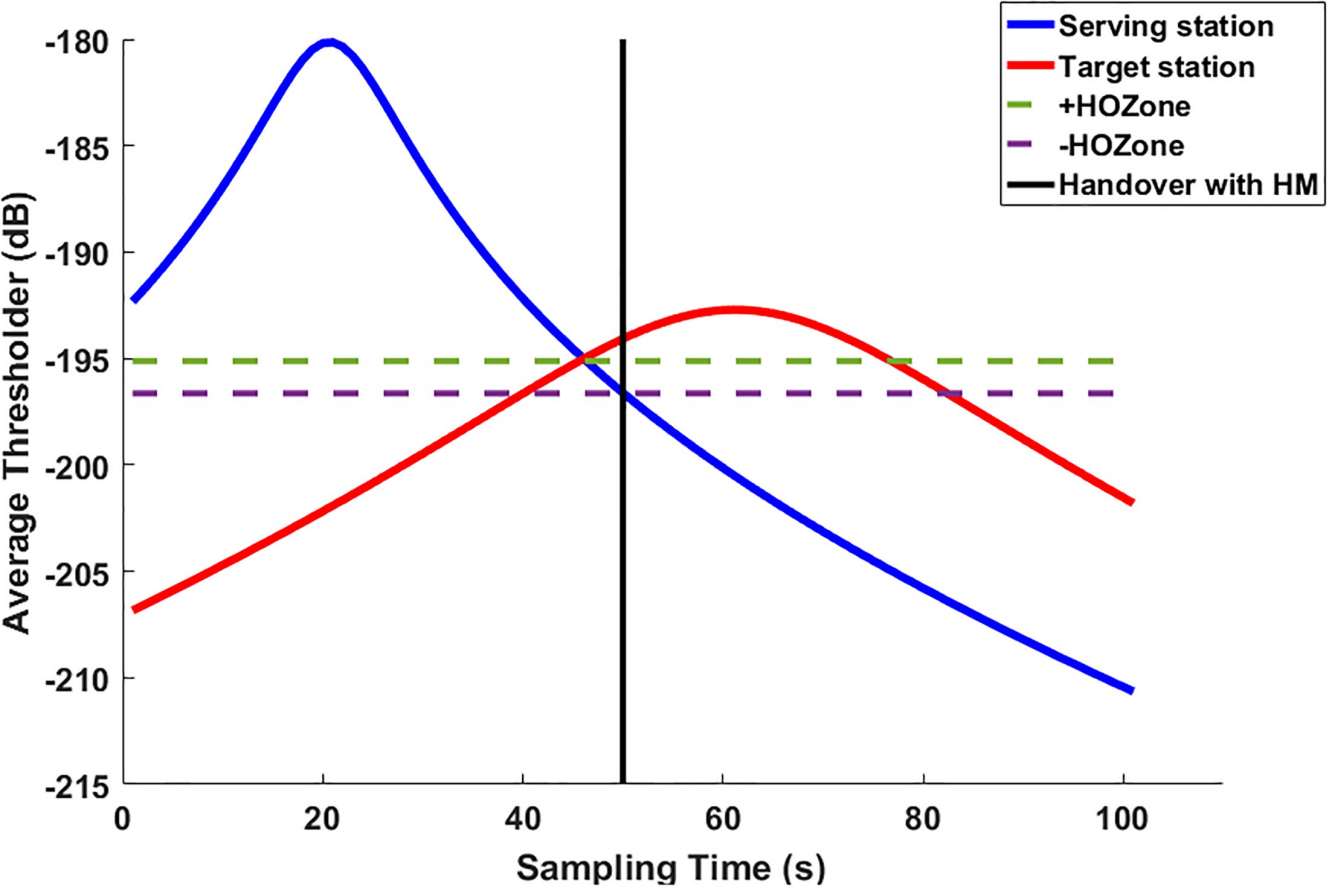
Predicted station and HO occurrence with HM.

**Fig 10:**
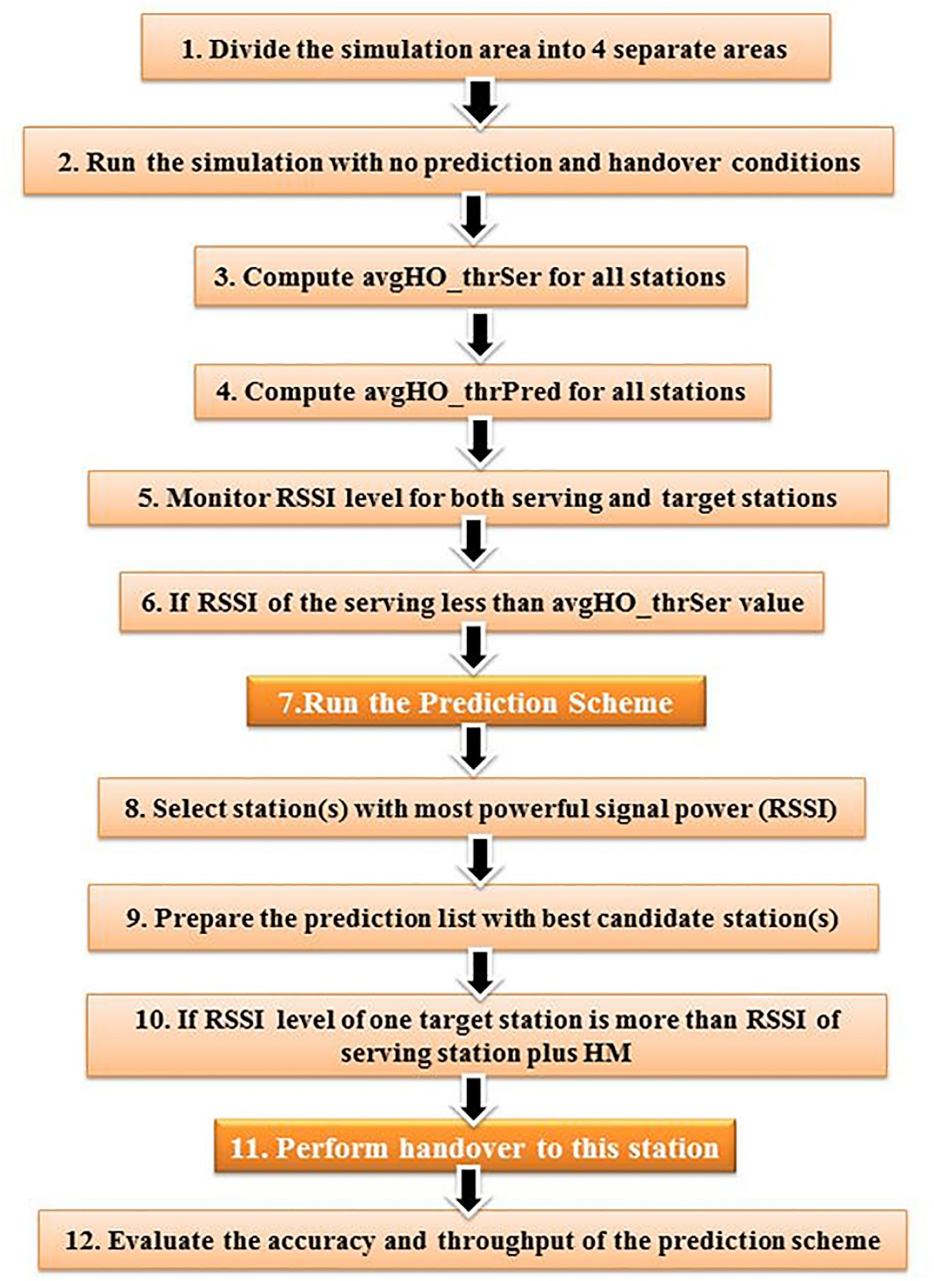
Proposed RSSI-based PHO simulation steps.

To avoid handover failure, the HM technique is combined in the handover decision where different values of HM are added according to Eq (11). The different values of HM enabled us to find the optimum situation for both predictions and handover occurrence. The positive effect of inclusion of the HM to the decision process is illustrated in Fig 9. The simulation steps of the proposed RSSI-based prediction scheme are summarized and displayed Fig 10.

$$S_{i}^{Pred}>S_{i}^{Ser}+HM$$ (11)

Where $S_{i}^{Pred}$ and $S_{i}^{Ser}$ are the signal strength of the predicted target station and the serving station correspondingly.

#### The multi-criteria prediction scheme

The nearby stations are divided into three categories: Powerful, Medium and Weak according to the radio frequency and the Quadrature Amplitude Modulation technique (QAM) [52] which widely used to modulate data signals on a carrier of radio communication devices where the QAM is different according to mobile kind.

As soon as the RSSI level of the serving station comes below the predefined threshold, the prediction process is started. The nearby stations are arranged according to the power level, however, the station with no available bandwidth will be named as a blocked station and consequently removed from the prediction process to prevent the MS from connecting to a station with no internet connection.

**Fig 11:**
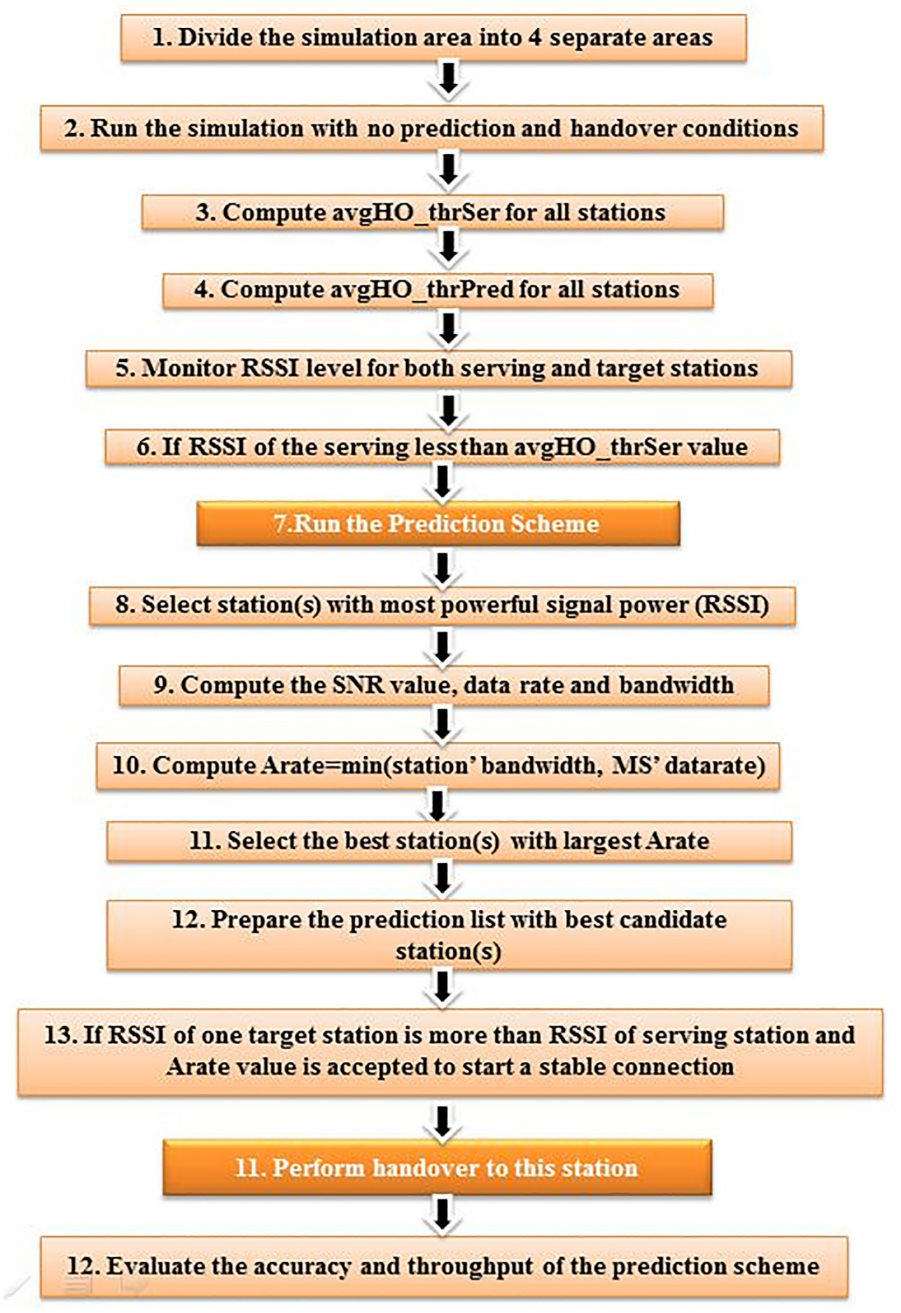
Multi-criteria PHO simulation steps.

The value of SNR is calculated according to the radio signals of all nearby stations where the prediction and the handover decision process are performed. The simulations steps of the proposed multi-criteria prediction handover scheme are illustrated in Fig 11.

### Simulation results

The proposed prediction schemes are being simulated using parameters shown in Table 2. The results are monitored and the output is evaluated. Both schemes are simulated and evaluated according to the same parameter and evaluating conditions.

#### RSSI-based prediction

**Fig 12:**
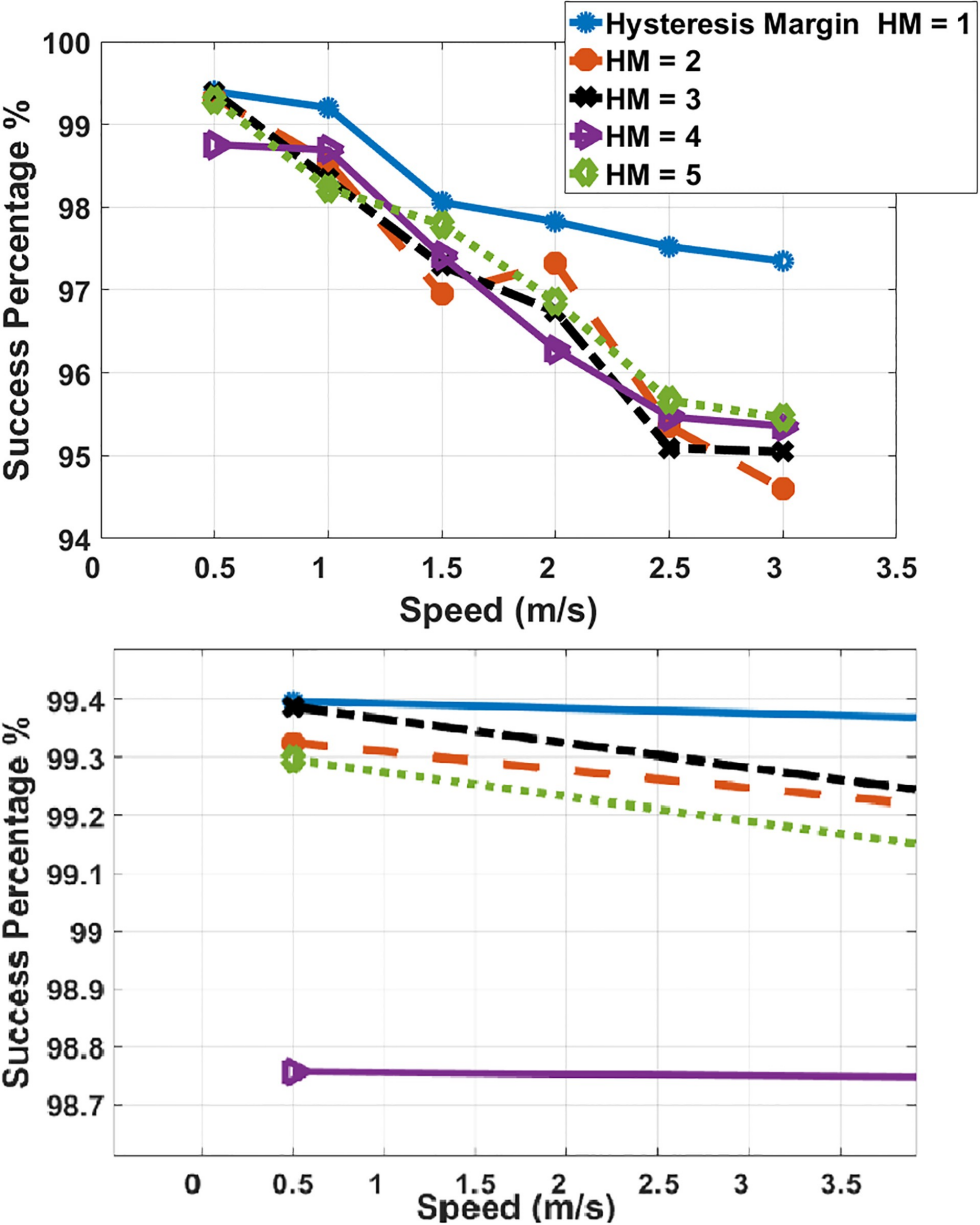
MS’ speed and success percentage.

The relation between the MS’ speed and accuracy percentage of the prediction scheme is illustrated in Fig 12 with HM = 1, 2, 3, 4 and 5 and MS’ speed = 0.5, 1, 1.5, 2, 2.5 and 3. The success percentage is 99% in a MS’ speed = 0.5 and with a HM = 1 which is a realistic result because the possibility of delivery from one station to another is reduced as the MS moves in low speed. In most cases, the predicted station is the station with the highest success ratio. Moreover, increasing MS’ speed will reduce the accuracy of the prediction scheme due to a large number of handovers that occur and the short time between each handover. Even though, the proposed scheme accomplishes a success ratio of more than 95% with high MS speed = 3 m/s and large HM value.

**Fig 13:**
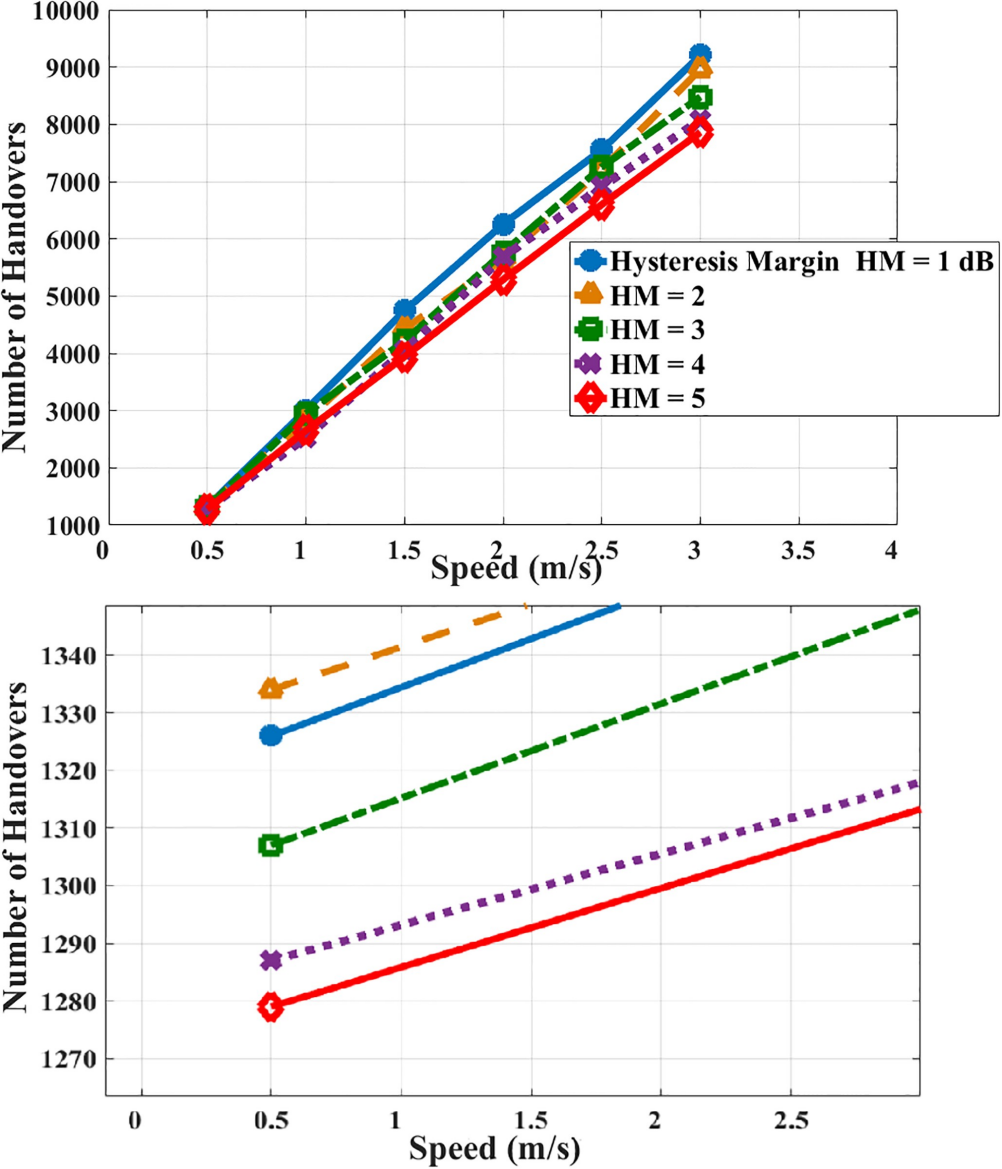
MS’ speed and number of handovers.

The relationship between the number of handovers occurred and different MS’ speeds is illustrated in Fig 13. It is observed that the relationship between the number of handovers and the speed of MS is proportional. The optimum case with the smallest number of handovers occurred at MS’ speed = 0.5 and HM = 1. Increasing the MS’ speed with HM = 1 increasing the number of handovers. We could reduce the number of handovers by increasing the value of HM.

**Fig 14:**
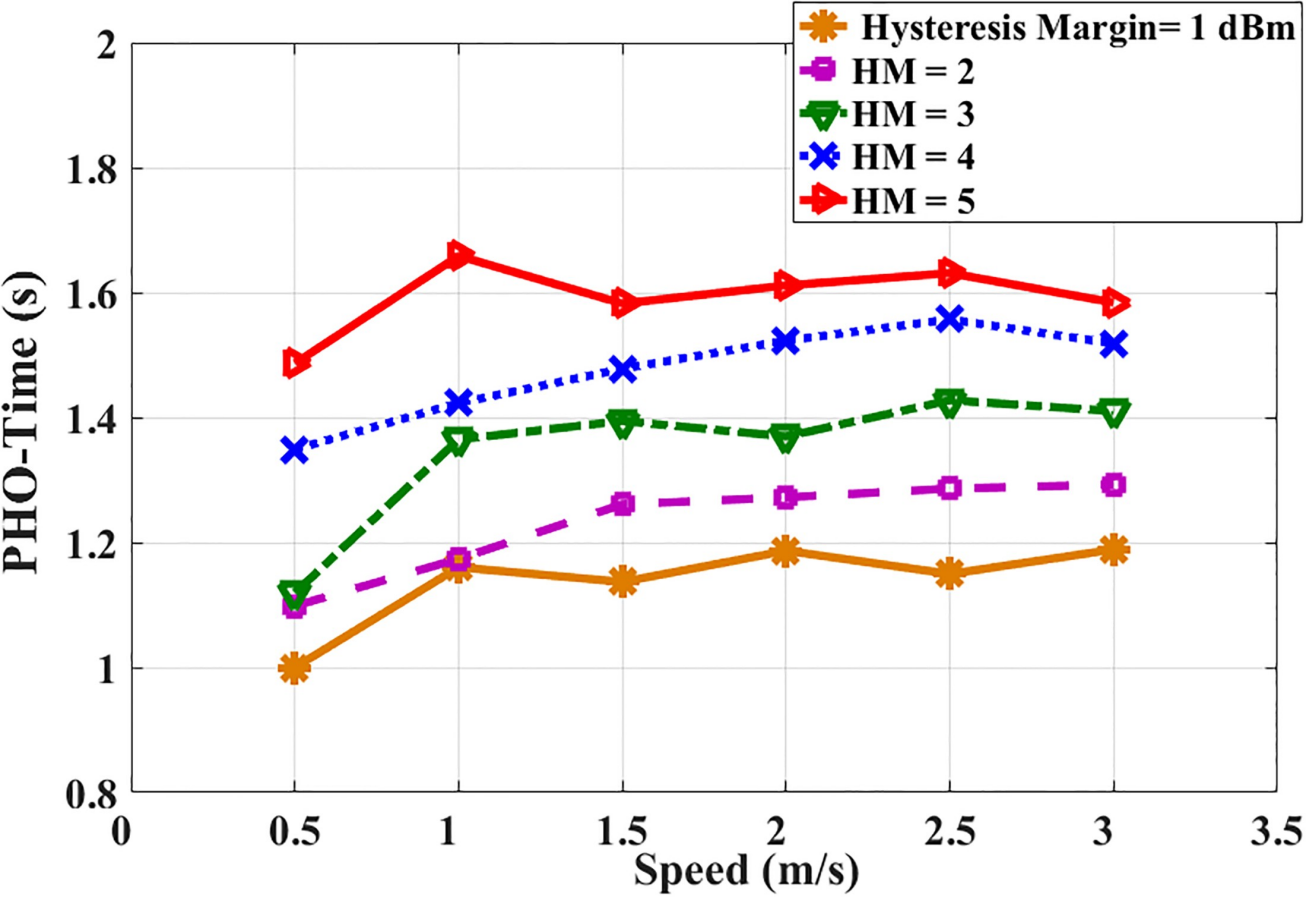
MS’ speed and time-PHO.

The simulation is performed without and with the proposed RSSI-based prediction scheme. The performance of the simulation with the proposed RSSI-based prediction scheme reduces the number of handovers of the MS with different speeds and several values of HM are displayed in Fig 14. It is clear that, the minimum value of Time-PHO is 1s while the maximum value is 1.8s which is relatively small. Furthermore, the PHO-time is enough to perform the prediction process, scanning process, comparing thresholds and arranging stations according to RSSI level where all of these processes required fractions of second.

#### Multi-criteria prediction scheme

**Fig 15:**
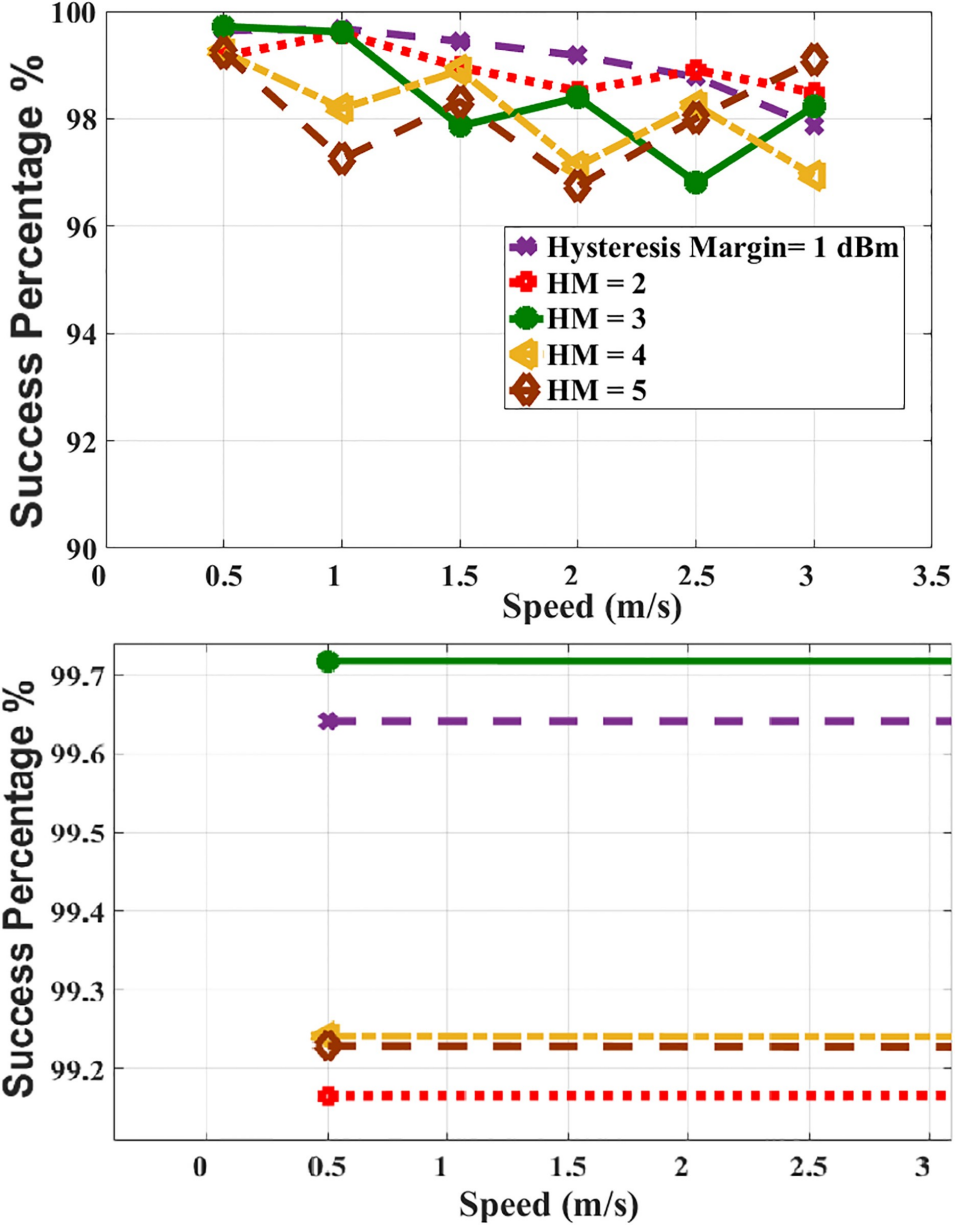
MS’ speed and success percentage.

The simulation is performed using the same parameter of the simulation of the RSSI-based prediction scheme. The MS’ speed is ranging from 0.5 m/s to 3 m/s and the values of the HM are ranging from 1 to 5. The effect of MS’ speed on the success percentage is illustrated in Fig 15. It is clear that the accuracy percentage is 96.5% to 99%. The variation in the percentages is according to the random movement manner of the MS and different values of HM where the success ratio doesn’t significantly change.

**Fig 16:**
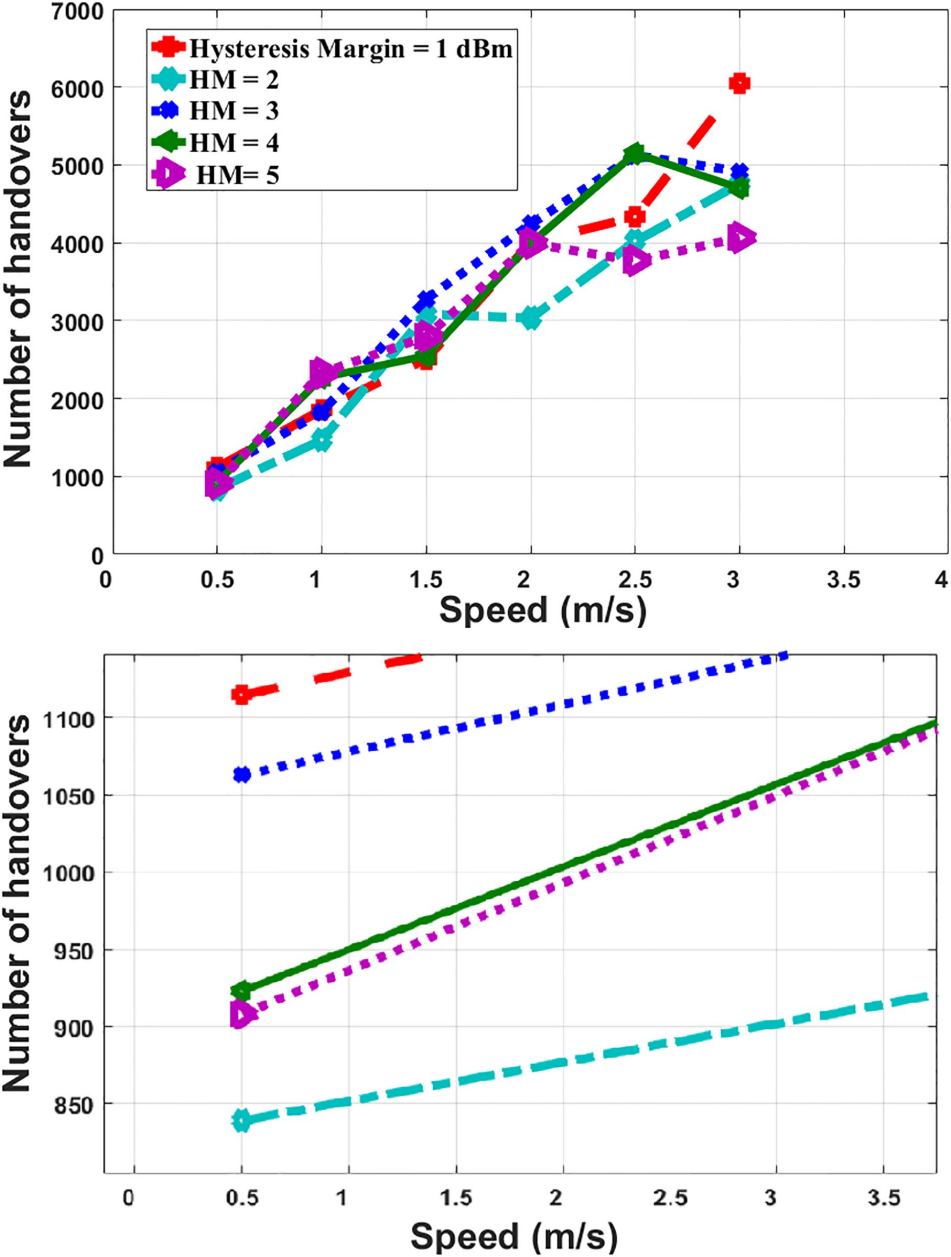
MS’ speed and number of handovers.

Fig 16 illustrates the number of handovers with various values of the HM and the MS’ speed. It is clear that, the number of handovers is increased when MS’ speed increased because the possibility of switching from one station to another station is increased.

By comparing the number of handovers with the RSSI-based prediction scheme, the maximum number is decreased from 9000 to 6000 due to using additional criteria in the prediction decision. The number of handovers occurred during the simulation period is monitored to recognize the effect of the proposed prediction scheme on the number of handovers an MS might do. The proposed scheme decreases the number of handovers by 95% when compared with the handover without any prediction.

**Fig 17:**
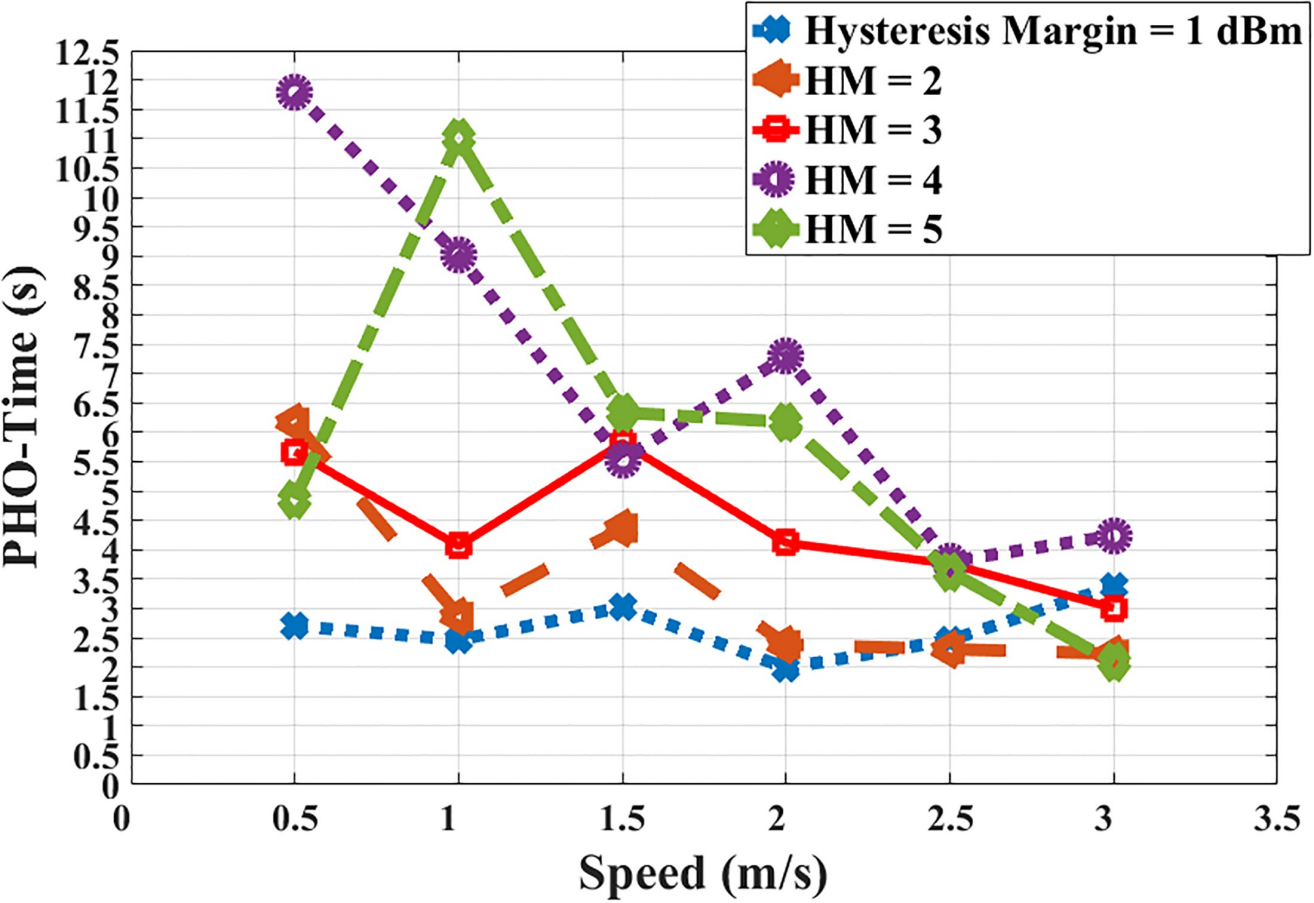
MS’ speed and PHO- time.

Fig 17 displays the relationship between the MS’ speed and the PHO-time. It is clear that, with the HM = 1, 2, and 3, the PHO-time is slightly changed and increased to 3s. For the values HM = 4 & 5, the change in PHO-time is increased to 6 s which is realistic.

## Comparison

To ensure the efficiency of the proposed schemes, we compared the performance of the proposed handover schemes with the existing schemes [20, 26, 28, 31–33] in terms of movement manner. The proposed handover prediction schemes, HOP, which utilizes two thresholds, outperformed all existing scheme. The performance of the proposed handover schemes is higher than the performance of the Bellavista et al. [20] by 30%. Becvar [26] achieved 45% for three neighboring stations in Manhattan-like road utilization. Becvar et al. [28] used two independent thresholds. Although they use the same concept, there are two main differences between our proposed schemes and the scheme in [28]. First, the TTP scheme which introduced by Becvar and his co-authors performs handover prediction only in WiMAX networks (Horizontal Handover) while the proposed HOP scheme designed to work with heterogeneous networks (Vertical Handover) as well as homogeneous networks. Second, both LTE and WiMAX networks have fixed locations and fixed certain areas for coverage which reduces the number of handovers. On the other side, the Wi-Fi AP is randomly located and their power are limited and variable which add more challenges to achieve successful handover.

**Fig 18:**
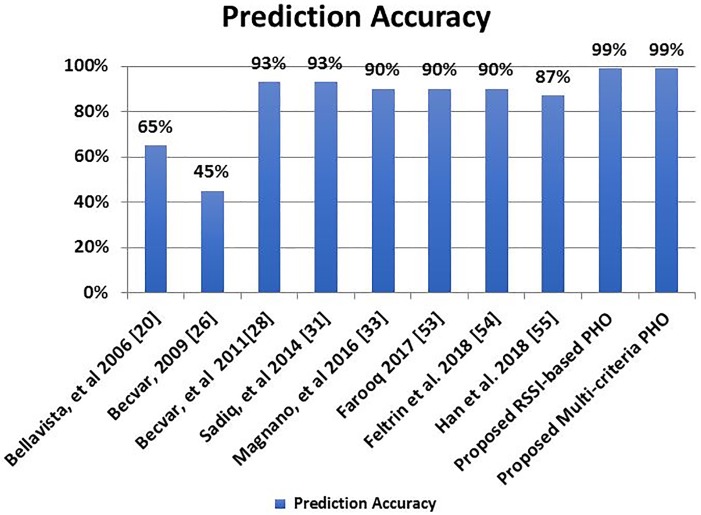
Maximum prediction accuracy.

Magnano et al. [33] used a KF-HMM scheme which decreased error in the prediction process and achieved a prediction accuracy 90%. For fair comparison, the proposed PHO schemes and the existing ones are based on the random movement of the MS where the obtained results are reported in Table 3 and visually displayed in Fig 18.

**Table 3:** Prediction accuracy.

Prediction Schemes	Prediction Accuracy	Improvement ratio Over existing Schemes
Bellavista, et al 2006 [20]	65%	34%
Becvar, 2009 [26]	45%	44%
Becvar, et al 2011 [28]	93%	6%
Sadiq, et al 2014 [31]	93%	6%
Magnano, et al 2016 [33]	90%	9%
Farooq 2017 [53]	90%	9%
Feltrin et al. 2018 [54]	90%	9%
Han et al. 2018 [55]	87%	12%
**Proposed RSSI-based PHO**	**99%**	**------**
**Proposed Multi-criteria PHO**	**99%**	**------**

Goudarzi et al. [32] presented the IRBF–FFA prediction scheme which is based on a fixed route and a constant MS’ speed with a specific number of stations. Their scheme achieved an accuracy rate between 97% and 99%. Based on the same conditions, the proposed PHO schemes achieve an accuracy rate equal to 100%. It is observed that the proposed PHO schemes outperformed all existing schemes whatever the movement manner is random or fixed.

The throughput improved ratio is computed and compared to existing schemes, depending on MS’ random mobility manner and in a fixed MS’ speed. The comparison is introduced in Table 4.

**Table 4:** Results for random mobility and fixed speed.

Prediction Schemes	Throughput Improved ratio
Wang et al. 2014 [6]	8.7%
Chen et al. 2014 [56]	11%
Tao et al. 2016 [57]	14%
Ahmad et al. 2018 [58]	14.9%
**Proposed RSSI-based PHO**	20%
**Proposed Multi-criteria PHO**	25%

## Conclusion

Two novel vertical handover prediction schemes based on channel features are proposed in this paper. The proposed PHO schemes are used with both heterogeneous as well as homogenous networks. Two new thresholds are defined and used in the proposed schemes to minimize the number of redundant handovers. The first proposed scheme is based on monitoring the RSSI values of all nearby stations. It achieves a prediction success with percentage 99% in the predefined surrounding areas using random MS’ movement manner. The second proposed prediction scheme is based on multi-criteria prediction and decision process. It used the SNR, the available bandwidth, the MS’ data rate and the RSSI values. It achieves a success rate 99% in the predefined simulation area.

The existing prediction schemes usually predicted only one station. The proposed PHO schemes create a prediction list which contains the stations with the best channel features for the next handover. This list is continuously updated to ensure the high success percentage. The performance of the proposed PHO schemes is compared with the performance of the existing prediction schemes in terms of random and fixed movement manner. The throughput improved ratio in the proposed protocol outperforms the ratio in the existing schemes in a random movement manner. The proposed PHO schemes outperformed all existing schemes for both kind of movement which ensure the superiority of the proposed PHO schemes over all existing schemes.

## Supporting information

S1 File This file contains simulation parameters. (DOCX)

S2 File This file is the table for results. (XLSX)

S3 File This is an APPENDIX which contains the sample code. (DOCX)
