# Supplementary material for: New vertical handover prediction schemes for LTE-WLAN heterogeneous networks
Source: PLoS One. 2019 Apr 17;14(4):e0215334. doi: 10.1371/journal.pone.0215334 (PMC6469805; doi:10.1371/journal.pone.0215334)
Supplement: S1 File — (DOCX) [file pone.0215334.s001.docx]

Simulation parameters

| **Parameter** | **Value** |
| --- | --- |
| Number of $BS_{s}$ | 19 |
| Number of $AP_{s}$ | 9, 12, 15, 17 |
| Number of $MS_{s}$ | 45, 60, 70, 90 |
| $BS$ transmitting power [dBm] | 46 |
| $AP$transmitting power [dBm] | 46 |
| $MS$ speed [m/s] | 0.5-3 |
| $MS$ gain [dBm] | 10 |
| $BS$ Frequency band [GHz] | 2.5 |
| $AP$ Frequency band [GHz] | 2.4 |
| Simulation duration [s] | 10800 |
| Scanning reporting period [s] | 1 |
| path loss model | Urban Macrocell |
| mobility model | PRWMM |
| Hysteresis margin HM [dB] | 1-5 |
