# Supplementary material for: New vertical handover prediction schemes for LTE-WLAN heterogeneous networks
Source: PLoS One. 2019 Apr 17;14(4):e0215334. doi: 10.1371/journal.pone.0215334 (PMC6469805; doi:10.1371/journal.pone.0215334)
Supplement: S3 File — (DOCX) [file pone.0215334.s003.docx]

## APPENDIX A: MOVEMENT.M

Function[Users]=Movement(No_Of_Users,BS,Users,Total_No_of_samples,Min_Speed,Max_speed)

for i = 1 : No_Of_Users

X_User = Users.Location(i,1);

Y_User = Users.Location(i,2);

No_Of_Points = 1;

Counter = 0;

while Counter < Total_No_of_samples

Temp_yq = [];

Temp_xq = [];

Temp_X_User = randi([ceil((min(min(BS.Location(:,1))))) ceil((max(max(BS.Location(:,1)))))],1,1);

Temp_Y_User=randi([ceil((min(min(BS.Location(:,2)))))ceil((max(max(BS.Location(:,2)))))],1,1);

Temp_Speed = randi([Min_Speed Max_speed],1,1);

Temp_Speed = Temp_Speed/2;

Temp_Distance = ((((X_User - Temp_X_User)^2) + ((Y_User - Temp_Y_User)^2))^0.5);

if Temp_X_User > X_User

Temp_x = [X_User , Temp_X_User];

Temp_y = [Y_User , Temp_Y_User];

Temp_xq = X_User : Temp_Speed : Temp_X_User;

Temp_yq = interp1(Temp_x,Temp_y,Temp_xq);

X_User = Temp_X_User;

Y_User = Temp_Y_User;

elseif Temp_X_User < X_User

Temp_x = [Temp_X_User , X_User];

Temp_y = [Temp_Y_User , Y_User];

Temp_xq = Temp_X_User : Temp_Speed : X_User;

R_Temp_Points = interp1(Temp_x,Temp_y,Temp_xq);

Temp_yq = fliplr(R_Temp_Points);

Temp_xq = fliplr(Temp_xq);

X_User = Temp_X_User;

Y_User = Temp_Y_User;

else

if Temp_Y_User > Y_User

Temp_x = [X_User , Temp_X_User];

Temp_y = [Y_User , Temp_Y_User];

Temp_yq = Y_User : Temp_Speed : Temp_Y_User;

Temp_xq = interp1(Temp_y,Temp_x,Temp_yq);

X_User = Temp_X_User;

Y_User = Temp_Y_User;

elseif Temp_Y_User < Y_User

Temp_x = [Temp_X_User , X_User];

Temp_y = [Temp_Y_User , Y_User];

Temp_yq = Temp_Y_User : Temp_Speed : Y_User;

R_Temp_Points = interp1(Temp_y,Temp_x,Temp_yq);

Temp_xq = fliplr(R_Temp_Points);

Temp_yq = fliplr(Temp_yq);

X_User = Temp_X_User;

Y_User = Temp_Y_User;

else

Temp_yq = 0;

end

end

if Temp_yq ~= 0

No_Of_Points = No_Of_Points + length(Temp_yq);

if No_Of_Points < Total_No_of_samples

z = 0;

k = 0;

while k ~= length(Temp_yq)

z = z + 1;

if Users.Location(i,1,z) == 0

k = k + 1;

Users.Location(i,1,z) = Temp_xq(k);

Users.Location(i,2,z) = Temp_yq(k);

end

end

else

z = 0;

k = 0;

while z < Total_No_of_samples

z = z + 1;

if Users.Location(i,1,z) == 0

k = k + 1;

Users.Location(i,1,z) = Temp_xq(k);

Users.Location(i,2,z) = Temp_yq(k);

end

end

end

end

Counter = No_Of_Points;

end

end

end

**APPENDIX B: OBSERVATION.M**

function [DataRate,Users,BS,AP,Total_HO_Counter,HO_Serving_Cells_Total] = Observation(DataRate,HO,Speed,HM,Observation_No_Of_Samples,No_Of_Users,Users,BS,AP)

Total_HO_Counter = 0;

RSSI_LTE_HO = [ ];

RSSI_Wifi_HO = [ ];

RSSI_LTE_Temp = Users.RSSI_LTE;

RSSI_Wifi_Temp = Users.RSSI_Wifi;

Serving_Cells_Wifi = zeros(No_Of_Users,1,2);

Users.Active_Serving_Cell = Users.Serving_Cells_Total;

Serving_Cells_LTE = Users.Serving_Cells_Total(:,:,[1 2]);

HO_Serving_Cells_Total = zeros(No_Of_Users,1,2);

User_HO_Counter_LTE = 0;

User_HO_Counter_Wifi = 0;

Counter_Ob = 0;

for j = 1 : No_Of_Users

Counter = 0;

for i = 2 : (Observation_No_Of_Samples+1)

condition = 1;

if Users.Active_Serving_Cell(j,1,3) ~= 0

idS = Users.Active_Serving_Cell(j,1,3);

Cell_type = 3;

else

idS = Users.Active_Serving_Cell(j,1,2);

Cell_type = 2;

end

while condition == 1

if max(RSSI_Wifi_Temp(j,:,i)) >= max(RSSI_LTE_Temp(j,:,i))

[RSSI_Temp idt] = max(RSSI_Wifi_Temp(j,:,i));

Free_BW = AP.BW_Limit(idt) - AP.BW(idt);

if idt ~= Users.Active_Serving_Cell(j,1,3)

if Free_BW >= DataRate.Min && RSSI_Temp >= AP.Signal_Limit

condition = 0;

Users.Serving_Cells_Total(j,i,:) = [RSSI_Temp 0 idt];

Users.Active_Serving_Cell(j,1,:) = [RSSI_Temp 0 idt];

AP.BW(idt) = AP.BW(idt) + min(Free_BW,Users.data_rate_wifi(j,idt,i));

DataRate.Serving(j,i) = min(Free_BW,Users.data_rate_wifi(j,idt,i));

if Cell_type == 3 && idS ~= 0

AP.BW(idS) = AP.BW(idS) - DataRate.Serving(j,i-1);

elseif Cell_type == 2 && idS ~= 0

BS.BW(idS) = BS.BW(idS) - DataRate.Serving(j,i-1);

end

Total_HO_Counter = Total_HO_Counter + 1;

Counter = Counter + 1;

Counter_Ob = Counter_Ob + 1;

User_HO_Counter_Wifi = User_HO_Counter_Wifi + 1;

Serving_Cells_Wifi(j,i,:) = [RSSI_Temp idt];

HO_Serving_Cells_Total(j,(Counter),:) = [Users.Serving_Cells_Total(j,i-1,1) RSSI_Temp];

Serving_Cells_LTE(j,i,:) = [0 0];

else

RSSI_Wifi_Temp(j,idt,i) = -10000;

end

else

if Free_BW >= DataRate.Min && RSSI_Temp >= AP.Signal_Limit

condition = 0;

AP.BW(idt) = AP.BW(idt) + min(Free_BW,(Users.data_rate_wifi(j,idt,i) - Users.data_rate_wifi(j,idt,i-1)));

DataRate.Serving(j,i) = DataRate.Serving(j,i-1) + min(Free_BW,(Users.data_rate_wifi(j,idt,i) - Users.data_rate_wifi(j,idt,i-1)));

Users.Serving_Cells_Total(j,i,:) = [RSSI_Temp 0 idt];

Users.Active_Serving_Cell(j,1,:) = [RSSI_Temp 0 idt];

Serving_Cells_Wifi(j,i,:) = [RSSI_Temp idt];

Serving_Cells_LTE(j,i,:) = [0 0];

else

RSSI_Wifi_Temp(j,idt,i) = -10000;

end

end

else

[RSSI_Temp idt] = max(RSSI_LTE_Temp(j,:,i));

Free_BW = BS.BW_Limit(idt) - BS.BW(idt);

if idt ~= Users.Active_Serving_Cell(j,1,2)

if Free_BW >= DataRate.Min & RSSI_Temp >= BS.Signal_Limit

condition = 0;

Users.Serving_Cells_Total(j,i,:) = [RSSI_Temp idt 0];

Users.Active_Serving_Cell(j,1,:) = [RSSI_Temp idt 0];

BS.BW(idt) = BS.BW(idt) + min(Free_BW,Users.data_rate_LTE(j,idt,i));

DataRate.Serving(j,i) = min(Free_BW,Users.data_rate_LTE(j,idt,i));

if Cell_type == 3 && idS ~= 0

AP.BW(idS) = AP.BW(idS) - DataRate.Serving(j,i-1);

elseif Cell_type == 2 && idS ~= 0

BS.BW(idS) = BS.BW(idS) - DataRate.Serving(j,i-1);

end

Total_HO_Counter = Total_HO_Counter + 1;

Counter = Counter + 1;

Counter_Ob = Counter_Ob + 1;

User_HO_Counter_LTE = User_HO_Counter_LTE + 1;

Serving_Cells_Wifi(j,i,:) = [0 0];

HO_Serving_Cells_Total(j,(Counter),:) = [Users.Serving_Cells_Total(j,i-1,1) RSSI_Temp];

Serving_Cells_LTE(j,i,:) = [RSSI_Temp idt];

else

RSSI_LTE_Temp(j,idt,i) = -10000;

end

else

if Free_BW >= DataRate.Min && RSSI_Temp >= AP.Signal_Limit

condition = 0;

Users.Serving_Cells_Total(j,i,:) = [RSSI_Temp idt 0];

Users.Active_Serving_Cell(j,1,:) = [RSSI_Temp idt 0];

BS.BW(idt) = BS.BW(idt) + min(Free_BW,Users.data_rate_LTE(j,idt,i) - Users.data_rate_LTE(j,idt,i-1));

DataRate.Serving(j,i) = DataRate.Serving(j,i-1) + min(Free_BW,Users.data_rate_LTE(j,idt,i) - Users.data_rate_LTE(j,idt,i-1));

Serving_Cells_Wifi(j,i,:) = [0 0];

Serving_Cells_LTE(j,i,:) = [RSSI_Temp idt];

else

RSSI_LTE_Temp(j,idt,i) = -10000;

end

end

end

if max(max(RSSI_Wifi_Temp(j,:,i)),max(RSSI_LTE_Temp(j,:,i))) < AP.Signal_Limit, condition = 0; break,end

end

end

end

Users.Counter_Observation(HO,Speed,HM) = Counter_Ob;

End

**APPENDIX C: GET_TARGET.M**

function [RSSI_T_Temp,Type_Of_Cell_t,idt,Free_BW] = get_target(HO_Zone_Thr,Temp_RSSI_Wifi_,Temp_RSSI_LTE_,BS,AP,Data_LTE,Data_Wifi)

RSSI_T_Temp = -1000;

Temp_RSSI_Wifi=[Temp_RSSI_Wifi_(find(Temp_RSSI_Wifi_>= HO_Zone_Thr))',find(Temp_RSSI_Wifi_ >= HO_Zone_Thr)',...

Data_Wifi(find(Temp_RSSI_Wifi_>= O_Zone_Thr))',AP.BW_Limit(find(Temp_RSSI_Wifi_ >= HO_Zone_Thr)) - AP.BW(find(Temp_RSSI_Wifi_ >= HO_Zone_Thr))];

Temp_RSSI_LTE=[Temp_RSSI_LTE_(find(Temp_RSSI_LTE_>=HO_Zone_Thr))',find(Temp_RSSI_LTE_ >= HO_Zone_Thr)',...

Data_LTE(find(Temp_RSSI_LTE_>= O_Zone_Thr))',BS.BW_Limit(find(Temp_RSSI_LTE_ >= HO_Zone_Thr)) - (BS.BW(find(Temp_RSSI_LTE_ >= HO_Zone_Thr)))];

Temp_RSSI_Wifi(:,5) = 3;

Temp_RSSI_LTE(:,5) = 2;

TN = vertcat(Temp_RSSI_LTE,Temp_RSSI_Wifi);

[val I] = sort(TN(:,1),'descend');

Total_neighbours = TN(I,:);

for i = 1 : size(Total_neighbours,1)

if Total_neighbours(i,4) >= Total_neighbours(i,3), Free_BW = Total_neighbours(i,3); RSSI_T_Temp = Total_neighbours(i,1); Type_Of_Cell_t = Total_neighbours(i,end); idt = Total_neighbours(i,2); break,end

end

if RSSI_T_Temp == -1000 && size(Total_neighbours,1) > 0

[val I] = max(Total_neighbours(:,4));

RSSI_T_Temp = Total_neighbours(I,1);

Type_Of_Cell_t = Total_neighbours(I,end);

idt = Total_neighbours(I,2);

Free_BW = min(Total_neighbours(I,3),Total_neighbours(i,4));

else

Temp_RSSI_Wifi=[Temp_RSSI_Wifi_(find(Temp_RSSI_Wifi_>= AP.Signal_Limit))',find(Temp_RSSI_Wifi_ >= AP.Signal_Limit)',...

Data_Wifi(find(Temp_RSSI_Wifi_>=AP.Signal_Limit))',AP.BW_Limit(find(Temp_RSSI_Wifi_ >= AP.Signal_Limit)) - AP.BW(find(Temp_RSSI_Wifi_ >= AP.Signal_Limit))];

Temp_RSSI_LTE=[Temp_RSSI_LTE_(find(Temp_RSSI_LTE_>= BS.Signal_Limit))',find(Temp_RSSI_LTE_>=BS.Signal_Limit)',... Data_LTE(find(Temp_RSSI_LTE_>= S.Signal_Limit))',BS.BW_Limit(find(Temp_RSSI_LTE_ >= BS.Signal_Limit)) - (BS.BW(find(Temp_RSSI_LTE_ >= BS.Signal_Limit)))];

Temp_RSSI_Wifi(:,5) = 3;

Temp_RSSI_LTE(:,5) = 2;

TN = vertcat(Temp_RSSI_LTE,Temp_RSSI_Wifi);

[val I] = sort(TN(:,1),'descend');

Total_neighbours = TN(I,:);

RSSI_T_Temp = Total_neighbours(1,1);

Type_Of_Cell_t = Total_neighbours(1,end);

idt = Total_neighbours(1,2);

Free_BW = min(Total_neighbours(1,3),Total_neighbours(1,4));

end

end
